# Phytochemical, In Vitro, In Vivo, and In Silico Research on the Extract of *Ajuga chamaepitys* (L.) Schreb.

**DOI:** 10.3390/plants13091192

**Published:** 2024-04-25

**Authors:** Elis Ionus, Verginica Schröder, Carmen Lidia Chiţescu, Laura Adriana Bucur, Carmen Elena Lupu, Denisa-Elena Dumitrescu, Liliana Popescu, Dragoș Paul Mihai, Octavian Tudorel Olaru, George Mihai Nițulescu, Rica Boscencu, Cerasela Elena Gîrd

**Affiliations:** 1 Faculty of Pharmacy, University of Medicine and Pharmacy “Carol Davila”, Traian Vuia 6, 020956 Bucharest, Romania; elis.ionus@drd.umfcd.ro (E.I.); liliana.popescu22@umfcd.ro (L.P.); octavian.olaru@umfcd.ro (O.T.O.); george.nitulescu@umfcd.ro (G.M.N.); rica.boscencu@umfcd.ro (R.B.); cerasela.gird@umfcd.ro (C.E.G.); 2 Faculty of Pharmacy, University of Constanţa “Ovidius”, 6 Căpitan Aviator Al. Șerbănescu Street, Campus C, 900001 Constanţa, Romania; laurabucur@univ-ovidius.ro (L.A.B.); clupu@univ-ovidius.ro (C.E.L.); denisa.dumitrescu@univ-ovidius.ro (D.-E.D.); 3 Faculty of Medicine and Pharmacy, “Dunărea de Jos”, University of Galaţi, 35 A.I. Cuza Street, 800010 Galați, Romania; carmen.chitescu@ugal.ro

**Keywords:** *Ajugae chamaepitys herbae extractum*, polyphenols, antioxidant activity, cytotoxic activity, UHPLC-HRMS/MS, molecular docking

## Abstract

The present study focuses on the chemical characterization of a dry extract obtained from the species *Ajuga chamaepitys* (L.) Schreb, evaluating its antioxidant properties, toxicity, and in silico profile. Quantitative analysis of the dry extract revealed a notable amount of phytochemical compounds: 59.932 ± 21.167 mg rutin equivalents (mg REs)/g dry weight, 45.864 ± 4.434 mg chlorogenic acid equivalents (mg ChAEs)/g dry weight and, respectively, 83.307 ± 3.989 mg tannic acid equivalents (TAEs)/g dry weight. By UHPLC-HRMS/MS, the following were quantified as major compounds: caffeic acid (3253.8 μg/g extract) and kaempherol (3041.5 μg/g extract); more than 11 types of polyphenolic compounds were quantified (genistin 730.2 μg/g extract, naringenin 395 μg/g extract, apigenin 325.7 μg/g extract, galangin 283.3 μg/g extract, ferulic acid 254.3 μg/g extract, p-coumaric acid 198.2 μg/g extract, rutin 110.6 μg/g extract, chrysin 90.22 μg/g extract, syringic acid 84.2 μg/g extract, pinocembrin 32.7 μg/g extract, ellagic acid 18.2 μg/g extract). The antioxidant activity was in accordance with the amount of phytochemical compounds: IC_50_DPPH = 483.6 ± 41.4 µg/mL, IC_50_ABTS^•+^ = 127.4 ± 20.2 µg/mL, and EC_50_FRAP = 491.6 ± 2 µg/mL. On the larvae of *Artemia* sp., it was found that the extract has a low cytotoxic action. In silico studies have highlighted the possibility of inhibiting the activity of protein kinases CDK5 and GSK-3b for apigenin, galangin, and kaempferol, with possible utility for treating neurodegenerative pathologies and neuropathic pain. Further studies are warranted to confirm the predicted molecular mechanisms of action and to further investigate the therapeutic potential in animal models of neurological disorders.

## 1. Introduction

Species of the genus *Ajuga* (e.g., *Ajuga chamaepitys* (L.), *Ajuga genevensis* (L.), *Ajuga reptans* (L.), and *Ajuga salicifolia* (L.)) are widespread in the spontaneous flora of Europe, Asia, Australia, North Africa, and North America and are associated with multiple therapeutic effects, mainly in Asia and Africa (e.g., analgesic, antibacterial, anti-inflammatory, anti-hypertensive, antioxidant, antipyretic, cardiotonic, hypoglycemic) [1,2].

*Ajuga chamaepitys* (L.) Schreb., called Ground Pine, is an annual plant that belongs to the Mediterranean area and is used for several effects in traditional medicine. Infusions prepared from its aerial parts are used to treat diarrhea, hemorrhoids, and various intestinal infections by drinking 3–4 cups of tea a day. Also, the plant has been found to have insecticide effects [3].

According to literature data, *Ajuga chamaepitys* (L.) Schreb, a species of the *Lamiaceae* family, contains flavonones (e.g., chrysoeriol 7-O-glucopyranoside, apigenin 7-O-rhamnopyranoside, isovitexin, orientin, flavonol) [4,5], polyphenols (e.g., gallic acid) [6], iridoids (e.g., acteoside, ajugoside, reptoside, 8-O-acetyl-harpagide, harpagide, 5-O-β-D-glucopyranosyl-harpagide, asperulosidic acid and diacetyl-asperulosidic acid) [7], diterpenes neoclerodans (e.g., ajugapitin and 14,15-dihydro-ajugapitin, 15-ethoxy-14-hydroajugapitin, ajugachin A, ajugachin B, ajugalaevigatic acid) [8,9,10,11], phytoecdysterones (e.g., cyasterone, 20-hydroxyecdysone, makisterone A) [12], and volatile compounds (e.g., α-pinene, β-pinene, β-phelandrene, germacrene D, (E)-phytol, ethyl linoleate, eucalyptol, limonene) [4,7,13,14].

Among the aforementioned phytochemicals, neo-clerodane diterpenoids are the characteristic and dominant constituents of the *Ajuga* species. In addition, iridoids (e.g., ajugol and ajugoside) are chemotaxonomic markers of the genus *Ajuga* [15].

In Romania, the species *Ajuga chamaepitys* (L.) Schreb. is dominant in the Dobrogea region (Dobrogea Gorges—44°29′16.5″ N–28°27′09.5″ E), where it is spread spontaneously in many areas, and is traditionally used in this region for analgesic and anti-inflammatory effects. We mention that the species that grows in this region of Romania has not been studied, and our research aims to form the basis of a larger study regarding the scientific foundation of therapeutic effects, correlated with the type of active chemical constituents.

In our previous study [16], the content of phytochemical compounds (flavonoids, phenolic acids, and total polyphenols) was analyzed in the aerial parts, flowers, leaves, roots, and stems collected in four flowering periods (May, June, July, August) using 70% ethanol (*v*/*v*). We found that plant materials collected in May contained the highest content in phytochemicals. Further analysis comparing extraction solvents—50% ethanol (*v*/*v*), 20% ethanol (*v*/*v*), and distilled water—and correlated with the results obtained on 70% ethanol extraction revealed that 50% ethanol (*v*/*v*) is the optimal solvent for extracting these phytochemical constituents, and the plant material collected in May had the most abundant content of phytochemicals. Although flowers are a plant product with an increased content of phytoconstituents, a small quantity of raw material is generated. We decided to continue the analyses on the aerial parts since they contain similar compounds, aiming to obtain a dry extract with potential therapeutic value. Extractive solutions in 50% ethanol, 20% ethanol, and water fall under the trend of implementing green technology and the use of green solvents [17,18].

The aim of this research was to evaluate the quality of an extract of *Ajuga chamaepitys* (L.) Schreb (spectrophotometric and UHPLC-HRMS/MS—ultra-performance liquid chromatography coupled with high-performance mass spectrometry resolution—analysis of polyphenols) obtained from the aerial parts of the species harvested from the spontaneous flora of Dobrogea Gorges, Romania. (44°29′16.5″ N–28°27′09.5″ E). For the projection of the following research, the extract was also subjected to in vitro evaluations (antioxidant assay by three methods: DPPH, ABTS^•+^, FRAP), in vivo evaluations (test of cytotoxicity on *Artemia salina* (L.) and *Daphnia* species and the teratogenic risk on *Daphnia magna* embryos), and in silico determinations (molecular docking studies for the potential of inhibiting the activity of protein kinases CDK5 and GSK-3b for apigenin, galangin, and kaempferol, with possible utility for treating neurodegenerative pathologies and neuropathic pain).

## 2. Results

### 2.1. Establishing the Identity and Quality of the Plant Raw Material

**Figure 1:**
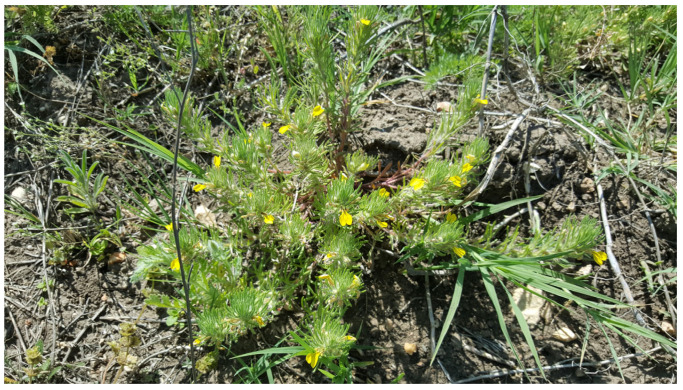
ACH located in Dobrogea Gorges, Romania.

*Ajugae chamaepitysis herba* (ACH) (Figure 1), called Ground Pine, is an annual plant that blooms in summer from May to August. It presents a thin, quadrangular, 10–30 cm long stem, its leaves are 1–4 cm long, with flowers which are up to 15 mm long. The corolla ranges from yellow to pale red, and it is 10–15 mm long, whereas its fruit appearance is a tetranucule, and the whole plant emits a pine scent when crushed [7,11,19].

The results for flavonoids, phenolic acids, and total polyphenols content for extractive solutions are shown in Table 1.

The results obtained guide the type of ethanol used (ethanol 50% (*v*/*v*)).

### 2.2. Obtaining and Characterizing the Dry Extract

A quantity of 52 g of dry extract *Ajugae chamaepitysis herbae extractum* (ACHE) was obtained with ethanol 50% (*v*/*v*), which is presented in the form of a green hygroscopic powder with a characteristic persistent smell. The extraction yielded 28.45% (the extract was kept in a desiccator).

#### 2.2.1. Spectrophotometric Determination of Flavonoids, Phenolic Acids, and Total Polyphenols

**Figure 2:**
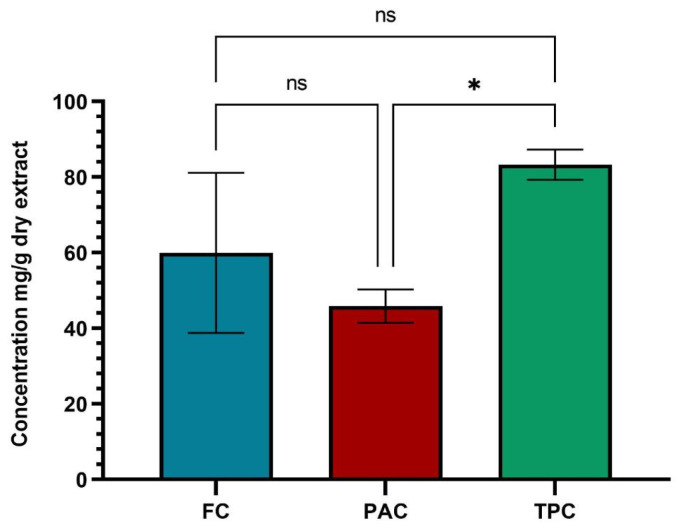
Results of the spectrophotometric determinations of the dry extract of ACHE. The data are represented as mean ± SD and analyzed using ANOVA with a Tukey post hoc test. * *p* < 0.05, ns: *p* > 0.05 no statistical differences.

The results of the quantitative analysis of flavonoids, phenolic acids, and total polyphenols for the selective dry extract are shown in Figure 2.

Thus, the following were obtained: 59.932 ± 21.167 mg rutin equivalents (mg REs)/g dry weight (dw), 45.864 ± 4.434 mg chlorogenic acid equivalents (mg ChAEs)/g (dw) and, respectively, 83.307 ± 3.989 mg tannic acid equivalents (TAEs)/g (dw).

#### 2.2.2. Identification and Quantification of Polyphenol Content Using UHPLC–HRMS/MS

**Figure 3:**
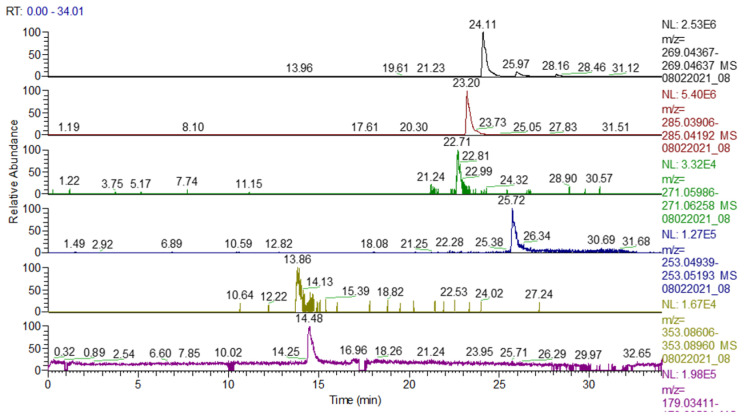
UHPLC-HRMS/MS chromatogram for ACHE in which the following were identified (top to bottom): apigenin (*m*/*z*: 269.04502, Rt: 24.11), kaempferol (*m*/*z*: 285.04049, Rt: 23.2), naringenin (*m*/*z*: 271.06122, Rt: 22.71), chrysin (*m*/*z*: 253.05066, Rt: 25.72), chlorogenic/neochlorogenic acid (*m*/*z*: 353.08783, Rt: 10.64/13.86), caffeic acid (*m*/*z*: 179.03501, Rt: 14.48).

**Figure 4:**
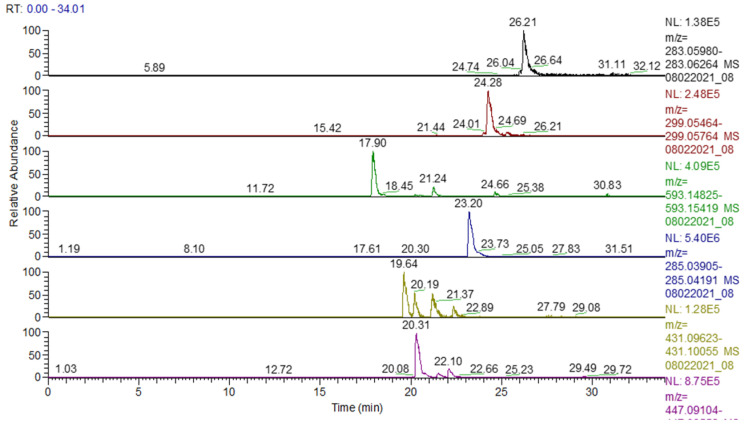
UHPLC-HRMS/MS chromatogram for ACHE in which the following were identified (top to bottom): biochanin A (*m*/*z*: 283.06122, Rt: 26.21), pratensein (*m*/*z*: 299.05614, Rt: 24.28), apigenin-7-O-glucosylglucoside (*m*/*z*: 593.1512176, Rt: 17.90), luteolin (*m*/*z*: 285.04049, Rt: 23.20), genistin (*m*/*z*: 431.09837, Rt: 19.64), vitexin (apigenin-8-C-glucoside)/isovitexin (*m*/*z*: 431.09839, Rt: 20.19/21.37), cynaroside (luteolin-7-O-glucoside) (*m*/*z*: 447.093284, Rt: 20.31).

A total of 19 polyphenolic compounds were identified: apigenin, kaempferol, naringenin, chrysin, genistin, 2′,6-dihydroxyflavone, caffeic acid, biochanin A, pratensein, kaempferol-3-O-rutinoside, kaempferol (or luteolin)-O-glucosides/isomers, vitexin (apigenin 8-C-glucoside)/isovitexin, apigetrin (apigenin-7-glucoside), cynaroside (luteolin-7-O-glucoside), apigenin 7-O-glucosylglucoside, hispidulin, luteolin, apigenin-7-O-glucuronide, and chlorogenic acid (Table 2, Figure 3 and Figure 4). These compounds are part of the group of flavonoids, isoflavones, and phenolic acids.

A total of 13 phytochemical compounds were quantitatively quantified (Table 3).

Following the analysis of these results, it was found that caffeic acid 3253.8 μg/g extract was assessed in significant quantities, followed by kaemferol 3041.5 μg/g extract, genistin 730.2 μg/g extract, naringenin 395 μg/g extract, apigenin 325.7 μg/g extract, galangin 283.3 μg/g extract, ferulic acid 254.3 μg/g extract, p-coumaric acid 198.2 μg/g extract, rutin 110.6 μg/g extract, chrysin 90.22 μg/g extract, syringic acid 84.2 μg/g extract, pinocembrin 32.7 μg/g extract, and ellagic acid 18.2 μg/g extract.

### 2.3. Evaluation of Antioxidant Activity

#### 2.3.1. Evaluation of the Scavenger Capacity of the DPPH Radical

The scavenger capacity of DPPH free radicals registered effects in accordance with the quantity of phytochemical compounds highlighted in the dry extract. Equations of the calibration curves of inhibition (%) vs. concentration (µg/mL) for the dry extract at 30 min, 60 min, and 90 min are represented in the Supplementary Materials (Figure S5a–c). It was observed that at the concentration of 1033 µg/mL, the inhibition was over 70%. IC_50_ values were calculated at 30 min being 529.4 µg/mL, at 60 min 472.4 µg/mL, and at 90 min 448.9 µg/mL, with an average of 483.6 ± 41.4 µg/mL.

#### 2.3.2. Evaluation of the Scavenger Capacity of the ABTS^•+^ Radical

To evaluate the scavenger capacity of the ABTS^•+^ radical, the equations of the calibration curves, inhibition (%) vs. concentration (µg/mL) for the dry extract (Figure S6), for a reaction time of 6 min, are frequently used (*n* = 3). It was observed that at a concentration of 409.6 µg/mL, the inhibition was over 80%. The IC_50_ values were calculated at 6 min (I) (Figure S6a), showing a value of 104 µg/mL, then 138.8 µg/mL at 6 min (II) (Figure S6b) and 139.3 µg/mL at 6 min (III) (Figure S6c), having an average of 127.4 ± 21.2 µg/mL.

#### 2.3.3. Evaluation of Antioxidant Capacity Based on Ferric-Reducing Capacity (FRAP)

For the assessment of the ferric reduction capacity, from Fe^3+^ to Fe^2+^, the equations of the calibration curves were graphically represented, concentration (µg/mL) vs. absorbance, for the dry extract (Figure S7), at 30 min (Figure S7a), 60 min (Figure S7b), and 90 min (Figure S7c). The absorbance values varied between 0.1459 (at a concentration of 40.8 µg/mL) and 0.9069 (at a concentration of 1020 µg/mL). EC_50_ values were calculated at 30 min, yielding a value of 491.6 µg/mL, 489.5 µg/mL at 60 min, and 493.4 µg/mL at 90 min, having an average of 491.6 ± 2 µg/mL.

**Figure 5:**
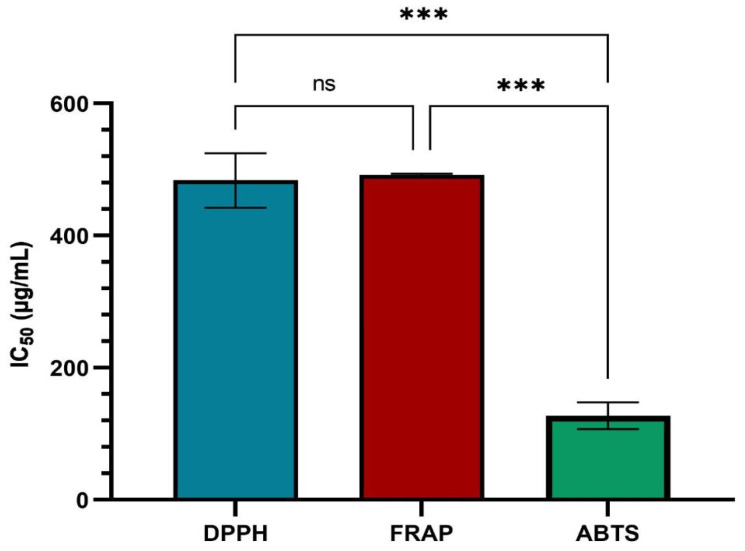
Antioxidant activity of ACHE. The data are represented as mean ± SD and analyzed using ANOVA with a Tukey post hoc test. *** *p* < 0.001, ns: *p* > 0.05 no statistical differences.

In Figure 5, antioxidant activity is statistically represented for the three methods used for ACHE.

### 2.4. Cytotoxic Assay

#### 2.4.1. Determination of the In Vivo Cytotoxicity of *Artemia* sp. Larvae (BSLA—Brine Shrimp Lethality Assay)

The results obtained by BSLA indicate significant effects of the extract at the tested concentration of 2600 µg/mL (LC_50_). The first records of mortality were from 557.85 µg/mL (LC_10_). The statistically analysis (Table 4) indicate statistically significant results and an important concentration–effects correlation (r^2^ = 0.9998, Supplementary Materials Figures S9 and S11).

LC_50_ mortality recorded at extract concentrations above 1000 µg/mL indicates a low cytotoxic effect. In the control samples, there was no larval mortality during the observation period. Observations allowed the identification of reduced, abnormal movements of larvae exposed to C_7_–C_11_ concentrations. These changes may be associated with the interaction of the extract (compounds) with cells that are not yet differentiated but play a role in contraction (myocytes).

#### 2.4.2. Daphnia Species Toxicity Assay

**Figure 6:**
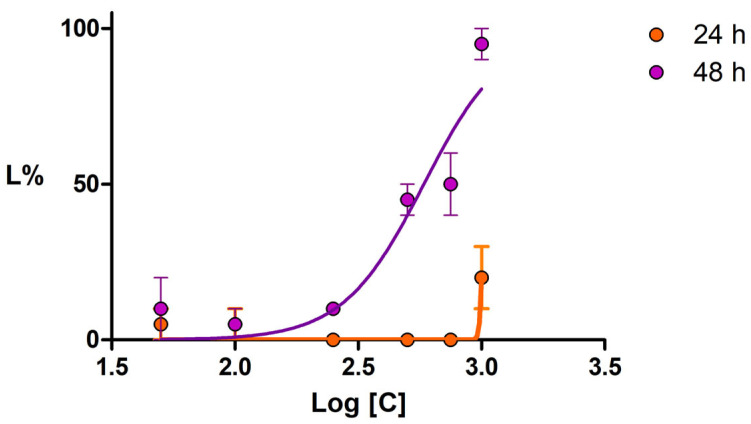
The lethality curves obtained after 48 h of exposure of *Daphnia pulex* to ACHE; error bars represent the SE of two replicates.

The extract was virtually non-toxic to *Daphnia magna* throughout the exposure period, with the highest recorded average lethality being 10%. On *Daphnia pulex*, the extract exhibited toxicity at high concentrations, indicating a greater sensitivity of this species to the extract. While the maximum lethality was 20% at 24 h of exposure, it ranged from 10% at the lowest concentration to 95% at the highest concentration at 48 h. Furthermore, the effect was concentration-dependent (r^2^ = 0.86). The LC_50_ at 48 h was 584.10 µg/mL, and the 95% confidence interval (CI 95%) was between 470.6 and 724.8 µg/mL, indicating low to moderate toxicity. The lethality curve is presented in Figure 6. The mobility of the crustaceans was significantly reduced at concentrations between 500 and 1000 µg/mL.

#### 2.4.3. *Daphnia magna* Embryo Developmental Assay

**Figure 7:**
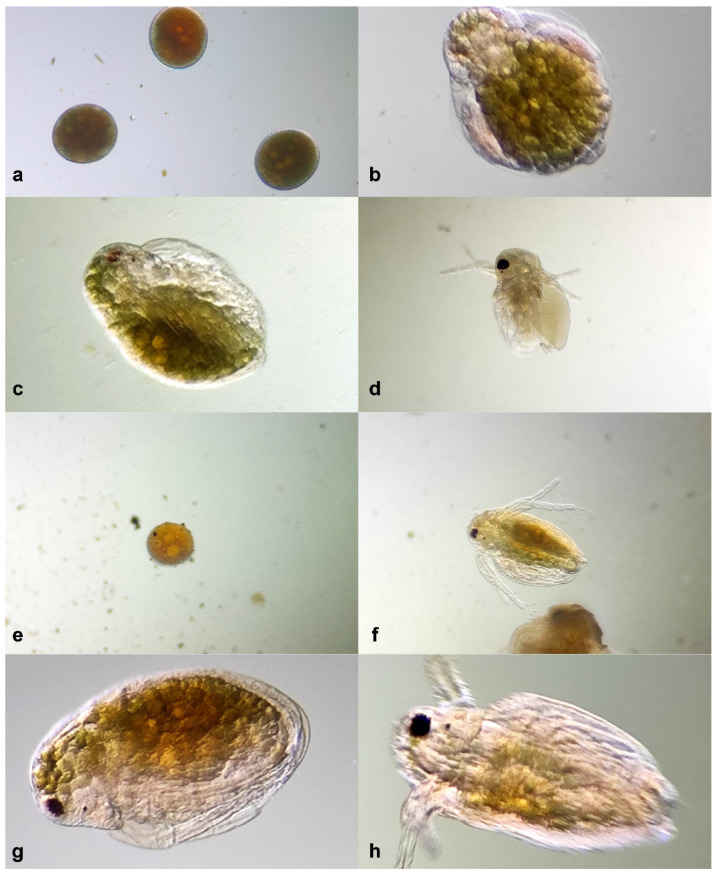
*Daphnia magna* embryo test: (**a**)—embryos at 0 h; (**b**)—intermediary larval stage treated with 500 µg/mL ACHE at 24 h; (**c**)—intermediary larval stage treated with 1000 µg/mL ACHE at 24 h; (**d**)—normal larvae treated with 500 µg/mL ACHE at 48 h; (**e**)—undeveloped egg treated with 1000 µg/mL ACHE at 48 h; (**f**)—larvae treated with 1000 µg/mL ACHE at 48 h; (**g**)—untreated control at 24 h; (**h**)—untreated control at 48 h.

At a concentration of 50 µg/mL, the developmental stage and viability of the embryos were similar to those of the untreated control. Thus, the formation of the compound eye, the antennae, the post-abdominal claw, and the shape of the rostrum and dorsal outline of the carapaces were similar to those observed in the untreated control. At 500 µg/mL, the viability was slightly lower than the control, with no abnormalities recorded in development, but there was a delay in the developmental stage. At the highest tested concentration of 1000 µg/mL, the extract affected the viability of the embryos, with the number of undeveloped embryos being three times higher than the control. The developed larvae exhibited reduced mobility, and the formation of the compound eye and antennae indicated a retardation effect (Figure 7).

Despite observing some retardation effects and a relatively high rate of toxicity on the *Daphnia* eggs, these effects were only registered at the highest concentration, which showed toxicity comparable to the control on the adult crustaceans. Therefore, the extract could be considered safe as it showed no evident teratogenic effects on the crustaceans.

The toxicity assays on *Daphnia* species revealed that the extract exhibited low to moderate toxicity, with effects being concentration-dependent. While higher concentrations affected *Daphnia pulex* significantly and delayed *Daphnia magna* embryo development, no teratogenic effects were observed, indicating relative safety at lower concentrations.

### 2.5. Prediction of Molecular Targets

Potential molecular targets were predicted for several phytochemicals (apigenin, galangin, genistein, kaempferol, naringenin, caffeic acid, ferulic acid, and p-coumaric acid), which were found in higher quantities based on UHPLC-HRMS/MS analysis. Following an analysis of prediction results, we selected CDK5 and GSK-3β as potential target candidates for the investigated polyphenols, both kinases being involved in the physiopathology of neurodegenerative diseases and neuropathic pain [20,21]. As shown in Table 5, apigenin was predicted as a CDK5 inhibitor with a 100% probability by SwissTargetPrediction, and with a 7.8% probability according to PASS. Moreover, apigenin had a max TC value of 61.54% when paired with known CDK5 inhibitors. Furthermore, apigenin had a 100% predicted probability of showing GSK-3β inhibitory activity according to SwissTargetPrediction algorithm and a 100% max TC value when paired with known GSK-3β inhibitors. After searching the ChEMBL database (10.1093/nar/gky1075), we found that apigenin was shown to inhibit both CDK5/p25 and GSK-3β activities, with similar potencies (IC_50_ values of 1.6 µM and 1.9 µM, respectively). Another interesting compound was kaempferol, for which SwissTargetPrediction algorithm predicted a 51.79% probability of inhibiting CDK5 and a 65.80% probability of inhibiting GSK-3β activity, while PASS algorithm yielded a 6.9% probability of acting as a GSK-3β inhibitor. A previous study showed that kaempferol inhibits CDK5/p25 with an IC_50_ value of 51 µM, but the activity on GSK-3β was not investigated. Furthermore, galangin was predicted with SwissTargetPrediction to have a 14.97% probability of inhibiting CDK5 and 16.61% of inhibiting GSK-3β, while PASS predicted a 9.80% probability of being active on CDK5. Interestingly, SEA calculated a 45.65% similarity between galangin and CDK5 inhibitors. Although naringenin was predicted as a potential CDK5 and GSK-3β inhibitor only by SwissTargetPrediction, previous studies showed that its glycoside, naringin, inhibits GSK-3β with an IC_50_ of 100 µM.

### 2.6. Molecular Docking

**Figure 8:**
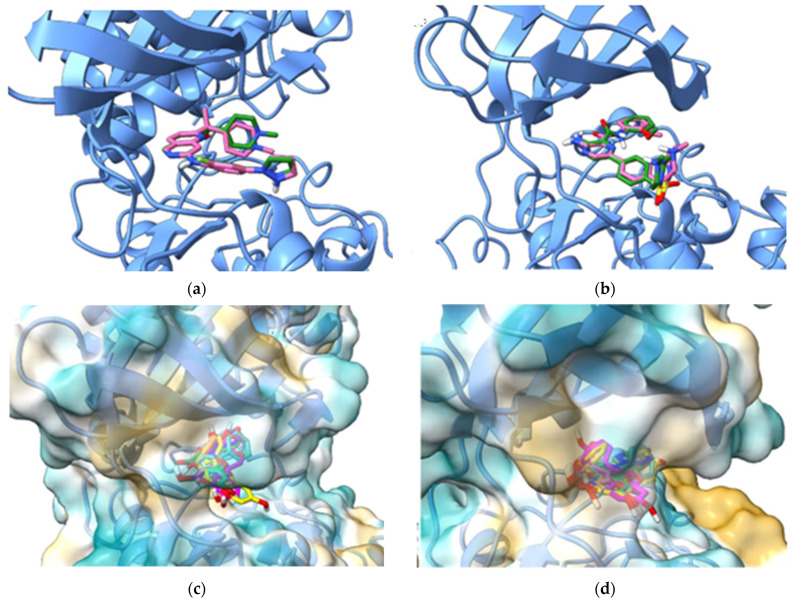
(**a**) Superposition of predicted pose (green) of CDK5 inhibitor on experimental conformation (pink); (**b**) Superposition of predicted pose (green) of GSK-3β inhibitor on experimental conformation (pink); (**c**) Docked poses of all screened phytochemicals into CDK5 ATP-binding site; (**d**) Docked poses of all screened phytochemicals into GSK-3β ATP-binding site.

Molecular docking studies were further performed to estimate the binding potential between the assessed polyphenols and the protein targets that were selected based on PASS, SwissTargetPrediction, and SEA predictions. Thus, the docking simulations were carried out against CDK5 and GSK-3β, both crystal structures being in active conformations (αCin architecture), since the αC-helix glutamate residues (Glu51 in CDK5 and Glu97 in GSK-3β) formed salt bridges with β3-strand lysine residues (Lys33 in CDK5 and Lys85 in GSK-3β). The docking protocol was validated by superposing the predicted poses of co-crystallized ligands on the experimental conformations (Figure 8a,b). The calculated RMSD values were 0.7023 Å for the CDK5 inhibitor and 1.2459 Å for the GSK-3β inhibitor, both values being lower than the 2.0 Å threshold.

**Figure 9:**
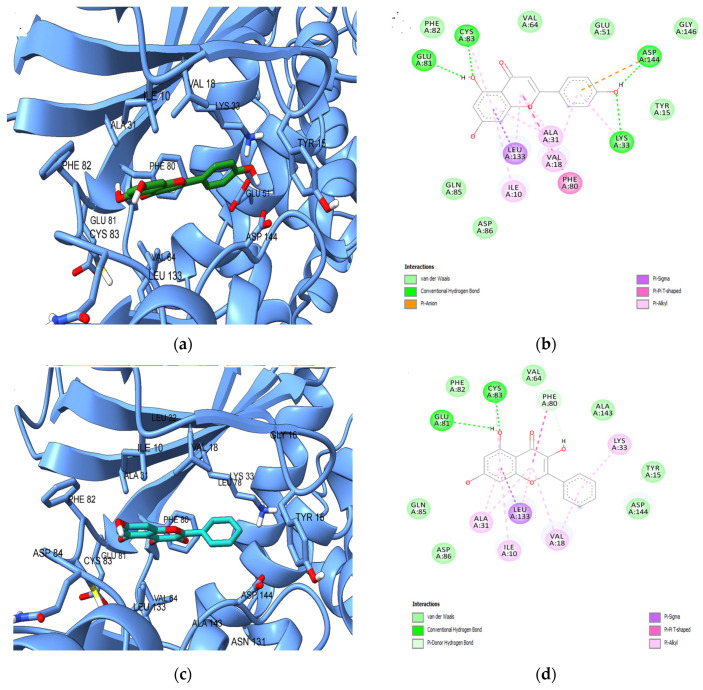
(**a**) Predicted conformation of apigenin in complex with CDK5; (**b**) 2D diagram illustrating predicted interactions between apigenin and CDK5 ATP-binding site; (**c**) Predicted conformation of galangin in complex with CDK5; (**d**) 2D diagram illustrating predicted interactions between galangin and CDK5 ATP-binding site.

The predicted binding energies and ligand efficiencies of the screened phytochemicals are shown in Table 6, and the docked poses of all compounds are illustrated in Figure 8c,d. According to the predictions, apigenin showed the lowest binding energy (−9.315 kcal/mol) after docking against the ATP binding site of CDK5, followed by naringenin (−9.232 kcal/mol) and galangin (−9.039 kcal/mol). After docking with GSK-3β, the lowest binding energies were obtained for galangin (−8.553 kcal/mol), apigenin (−8.551 kcal/mol), and naringenin (−8.537 kcal/mol). We further chose to discuss the predicted molecular interactions between apigenin (as a positive control) and both protein kinases, since the flavonoid was confirmed as a CDK5 and GSK-3β inhibitor according to the data deposited in ChEMBL database. As shown in Figure 9a,b, the hydroxyphenyl sub-structure of apigenin formed a hydrogen bond with β3-strand Lys33 and Asp144 within the conserved kinase DFG motif, while the 7-hydroxy moiety engaged in hydrogen bonding with Cys83 within the hinge and Glu81. Furthermore, the catalytic Asp144 interacted with the phenyl moiety of apigenin also through pi-anion interactions. Moreover, several hydrophobic interactions (pi-sigma, pi-pi T-shaped, pi-alkyl) are formed between the benzopyran-4-one scaffold and residues Ile10, Val18, Ala31, Phe80, and β7-strand residue Leu133, which is the “floor” of the adenine binding pocket.

Based on the results of target prediction approaches and molecular docking simulations, we further chose to discuss the predicted interactions between the potential CDK5 inhibitor galangin and the ATP-binding site (Figure 9c,d). Similar to apigenin, galangin engaged in hydrophobic interactions with Ile10, Val18, Ala31, Phe80, and Leu133 and formed hydrogen bonds with Glu81 and Cys83. However, the non-hydroxylated phenyl moiety formed only pi-alkyl interactions with Lys33, while a pi-donor hydrogen bond is formed between Phe80 and the 3-hydroxy radical, which is absent in apigenin.

**Figure 10:**
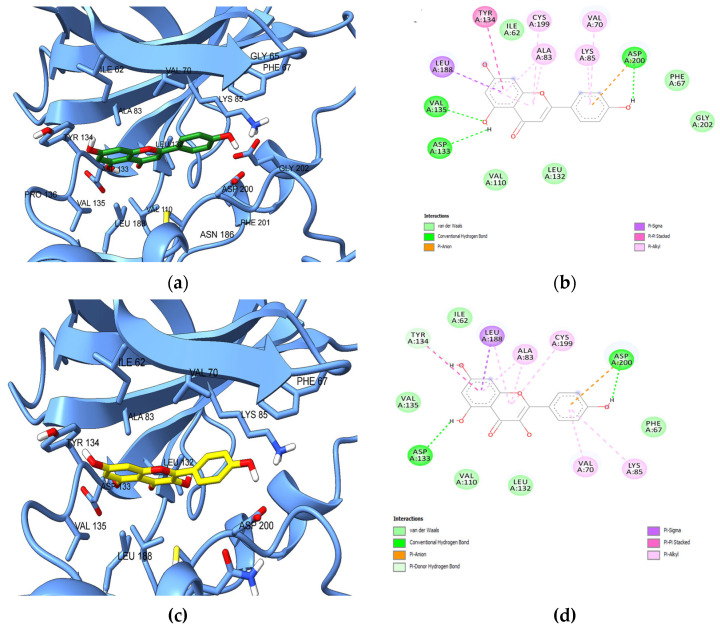
(**a**) Predicted conformation of apigenin in complex with GSK-3β; (**b**) 2D diagram illustrating predicted interactions between apigenin and GSK-3β ATP-binding site; (**c**) Predicted conformation of kaempferol in complex with GSK-3β; (**d**) 2D diagram illustrating predicted interactions between kaempferol and GSK-3β ATP-binding site.

Next, we analyzed the predicted interactions between the positive control apigenin and the ATP binding site of GSK-3β (Figure 10a,b). The 5-hydroxy radical of apigenin formed hydrogen bonds with adenine pocket residues Val135 and Asp133, while the hydroxy-phenyl radical engaged in hydrogen bonding and pi-anion interactions with catalytic Asp200 within the DFG motif and in pi-alkyl interactions with Val70 and catalytic Lys85. Furthermore, hydrophobic interactions (pi-sigma, pi-pi stacked, pi-alkyl) were formed between the benzopyran-4-one scaffold and Ala83, Tyr134, hinge residue Cys199 and “floor” residue Leu188. Similarly, kaempferol, which was predicted as a potential GSK-3β inhibitor by PASS and SwissTargetPrediction, formed the same interactions as apigenin with Asp200 within DFG motif, catalytic Lys85, “floor” residue Leu188, hinge residue Cys199, and residues Val70m, Ala83, Asp133, and Tyr134. However, no hydrogen bonds were formed with Val135, but a pi-donor hydrogen bond was predicted between the 7-hydroxy radical and Tyr134. Nonetheless, the 3-hydroxy radical that is absent in apigenin showed no interactions with the ATP-binding site.

## 3. Discussion

The flora in the Dobrogea Region, Romania, includes approximately 2.000 taxa, representing approximately 50% of Romania’s flora. The particular flora is impressive from a scientific point of view considering that it includes a large number of species, some of which are rare plants (approximately 200), among which we mention the species protected at the European level: *Achillea clypeolata*, *Centaurea jankae*, *Campanula romanica*, *Moehringia jankae*, and *Paeonia tenuifolia*. The vegetation consists of numerous continentals, Balkan, sub-Mediterranean, and Mediterranean species [22]. We chose to study the species *Ajuga chamaepitys* (L.) Schreb, known for its traditional uses in the Dobrogea region but not scientifically documented.

Our research aimed to evaluate the quality of a lyophilized *Ajuga chamaepitys* (L.) Schreb. extract for its antioxidant, cytotoxic actions and to predict its potential neuropathic pain relief properties.

Caffeic acid is found naturally in various foods, beverages, nuts, herbs, vegetables, fruits, and oils [23]. It has been studied from a pharmacological point of view, demonstrating its antioxidant, immunomodulatory, antimicrobial, antiviral, neuroprotective, antiproliferative, and anti-inflammatory capacity [24,25,26,27,28,29,30].

The analysis of the data obtained by us compared with the existing literature is limited due to the small number of studies on this species and the different methods of extraction and analysis. In previous reports on *Ajuga* species from Romania, *Ajuga laxmanii* (L.), the following polyphenolic compounds were isolated in the methanol extract [31] using an optimized HPLC/MS method: rutin 6721.49 μg/g dw (dry weight), isoquercitrin 636.1 μg/g dw, apigenin 126.53 μg/g dw, luteolin 88.24 μg/g dw, quercitrin 36.5 μg/g dw, chlorogenic acid (caffeic acid derivative) 19.33 μg/g dw. Analyzing these data, rutin showed low values in our extract, 110.6 μg/g; instead, a higher value of caffeic acid (3253.8 μg/g) was quantified. Ghiţă et al. conducted a comparative phytochemical study between the species *Ajuga reptans* (L.) and *Ajuga genevensis* (L.), both collected from the spontaneous flora of Romania, where identified compounds such as chlorogenic acid, caffeic acid, apigenol, and luteolin-7-O-glucoside [32] were also present in our extract. Toiu et al. (2017) analyzed the methanolic extract from the flowers of the species *Ajuga reptans* (L.) collected from the spontaneous flora of Cluj, Romania. The research group identified and quantified the polyphenols by LC-MS, recording the majority component caffeic acid 34.39 μg/g dw (dry weight), followed by luteolin 26.13 μg/g dw, p-coumaric acid 26.02 μg/g dw, apigenin 23.56 μg/g dw, ferulic acid 20.15 μg/g dw, and quercitrin 4.97 μg/g dw [33]. Our analysis recorded higher amounts of these compounds, except for quercitrin, which was not detected, and luteolin, whose isomer, kaempferol, was detected in our extract.

The phytochemical analysis of extracts (from aerial parts) of *Ajuga reptans* (L.) and *Ajuga genevensis* (L.) collected from the spontaneous flora of Romania identified hyperoside, isoquercitrin, rutin, quercitrin, p-coumaric acid, ferulic acid, apigenin, and luteolin, whereas caffeic acid was detected only in *Ajuga genevensis* extract [34]. In our extract, caffeic acid was the major component, the amount being 3253.8 μg/g.

Our findings showed that ACHE has significant antioxidant activity, confirming the results of other researchers. Moreover, even though the free radical scavenging effect of ACHE is several folds lower than the reference antioxidant activity (IC_50_ DPPH extract = 529.4 µg/mL higher than IC_50_ ascorbic acid = 16.5 µg/mL), we can observe that the Pearson coefficients (Supplementary Materials Table S3) regarding the DPPH values and the concentrations of chemical compounds that generate the effect reached the highest significant level (DPPH vs. FC: r = 0.9959, *p* < 0.05; DPPH vs. PAC: r = 0.9991, *p* < 0.001; DPPH vs. TPC: r = 0.9704, *p* < 0.05). These results highlighted the precision of the correlation between the variables as well as the high degree of accuracy of the DPPH method in contrast to the other antioxidant techniques. Turkoglu et al. (2010) concluded that the antioxidant effect of an aqueous extract of *Ajuga chamaepitys* (L.) ssp. *euphratica* was superior to extracts obtained with methanol and chloroform [35]. The aerial parts of *Ajuga chamaepitys* (L.) Schreb. from Iran, extracted with methanol of various concentrations, had the best inhibition on testing the DPPH free radical scavenging activity [4]. Similar results were obtained for the acetone extract of *Ajuga chamaepitys* ssp. *chamaepitys* collected from Serbia [6]. In another research on the polar extracts (in ethanol and in water) and the essential oil of *Ajuga chamaepitys* (from *herba*), collected from central Italy, it was found that the antioxidant activity of the polar extracts was higher than that of the essential oil, and moderate compared with that of Trolox [7]. Thus, our results are consistent with those reported in the literature, demonstrating that ACHE has comparable antioxidant properties with extracts obtained from *Ajuga chamaepitys* (L.), collected from different geographical areas.

Testing of *Artemia* species revealed no effects on cell division. Evaluation at 24–30 h after exposure showed that all larvae passed into larval stage II (microscopic observations). This passage is made by molting, a phenomenon that requires repeated divisions; therefore, the cellular processes specific to the division were not disturbed by the composition of the extract. The LC_50_ value, which expresses the mortality of 50% of the subjects, was higher than 1000 μg/mL, indicating low cytotoxic action.

The toxicity evaluation on *Daphnia magna* and *Daphnia pulex* revealed different sensitivities to the *Ajuga* extract. *D. magna* demonstrated significant resistance, with negligible lethality caused by the extract. In contrast, *D. pulex* exhibited high sensitivity at elevated concentrations, particularly after a 48h exposure period. Although other studies showed that overall, the differences in responses between *D. magna* and species considered more sensitive, such as *D. pulex* or *Ceriodaphnia dubia*, were not substantial; the variability of each population could be an important factor that influences the response of the crustaceans [36,37]. Our research found the use of both species advantageous because each yielded unique sensitivity responses, thus serving as a valuable tool for identifying potential toxic effects induced by plant extracts. However, toxicity was evident only at concentrations significantly higher than those typically used in phytotherapy.

The embryo test on *D. magna* embryos can be used to identify potential teratogenic effects [38,39]. In our research, the *D. magna* embryo developmental assay further elucidated the impact of the extract. Thus, between 50 and 500 µg/mL, the developmental stages and viability were slightly lower or comparable with the untreated control, whereas at 1000 µg/mL, a very high concentration, the viability was reduced, the development of the larvae was delayed, and abnormal modifications were observed.

*Ajuga chamaepitys* (L.) Schreb from Dobrogea, Romania, is known in traditional medicine for its anti-inflammatory and analgesic effects, and therefore, we focused our research on predicting its potential use in neuropathic pain. Jaffal et al. (2019) showed that the methanolic extract of *Ajuga chamaepitys* from Jordan mediated the antinociceptive activity in experimental models on mice. Mediation of the analgesic effect by the involvement of an opioid receptor was evaluated by administration of an opioid receptor antagonist (naloxone), which resulted in antagonism of the analgesic effect of *Ajuga chamaepitys* extract. The results showed that the methanolic extract induced significant, dose-dependent analgesic effects [5]. Similar results were obtained by Khanavi et al. (2014) using extracts from *Ajuga chamaecistus ssp. Tomentella* on mice, showing a significant analgesic effect in the chronic phase, especially the extracts from water, hexane, and ethyl ether [40]. The mechanism underlying this phenomenon is thought to be the inhibition of the biosynthesis of proinflammatory prostaglandins, and the main compound responsible for this action would be 8-acetylharpagide [40], a compound also found in *Ajuga chamaepitys* (L.) Schreb. Although other mechanisms can be responsible for the analgesic and anti-inflammatory effects, these observations support the traditional use of *Ajuga* species for pain and other inflammatory diseases. Thus, we predicted potential molecular targets for several polyphenols, which we identified in the ACHE. Following the predictions, we identified serine/threonine protein kinases CDK5 and GSK-3β as potential therapeutic targets for the flavonoids apigenin, galangin, and kaempferol. Molecular docking studies further supported the interaction potential between apigenin and the ATP-binding site of both kinases, between galangin and CDK5, and between kaempferol and GSK-3β, respectively. According to bioassay data deposited in the ChEMBL database, apigenin inhibits CDK5 and GSK-3β at micromolar concentrations, while kaempferol inhibits CDK5. Since both protein kinases are dysregulated in several neurodegenerative diseases (e.g., tauopathies) and in neuropathic pain [20,21], the investigated extract could show therapeutic effects in such disorders through dual inhibition of CDK5 and GSK-3β. One limitation of the present study is the lack of experimental validation of the computational results. Further studies are warranted to support the present findings and to evaluate the therapeutic efficacy of *Ajuga chamaepitys* (L.) Schreb extract in animal models of neuropathic pain and other neurological conditions.

## 4. Materials and Methods

### 4.1. Plant Material

The aerial parts of the species *Ajuga chamaepitys* (L.) Schreb. were collected in May 2022 from the spontaneous flora of the Dobrogea region, Romania (44°29′16.5″ N–28°27′09.5″ E), in the morning, in dry and sunny weather. The species was identified by Prof. PhD Laura Bucur from the Department of Pharmacognosy, Phytotherapy and Phytochemistry, and Assoc. Prof. PhD Verginica Schröder from the Department of Celullar Biology and Genetics, Faculty of Pharmacy, “Ovidius” University Constanţa, and several specimens are in the collection of the Pharmacognosy Laboratory. The solvents and experimental conditions used in this study are presented at each step. Also, we mention that this species’ spread in the Dobrogea area does not indicate problems of endangering the plant raw material; the species is adapted to the pedoclimatic conditions of this region of the country, and it spreads spontaneously and is very abundant.

Our research was carried out in several stages, as follows: (1) establishing the quality of the plant raw material; (2) obtaining and chemical characterization of the dry extract by spectrophotometric and chromatographic methods (UHPLC-HRMS/MS); (3) evaluation of the antioxidant capacity; (4) determination of cytotoxicity in vivo on the larvae of *Artemia* sp. (BSLA) and *Daphnia*; (5) determination of the teratogenic potential by the embryo test on *Daphnia magna* embryos; (6) molecular docking predictions; and (7) potential interactions between the quantified compounds and types of enzymes. All the results were statistically processed.

### 4.2. Establishing the Quality of the Plant Material

In this stage of work, we verified the quality of the aerial parts of the plant (dried in laboratory conditions) by spectrophotometric determination of three types of phytochemical compounds (flavonoids, phenolic acids, and total polyphenols). Extractions were performed separately for each type of sample in 50% ethanol, 20% ethanol, and water. Extracts were prepared by refluxing for 30 min. One g of herbal product (*herba*—noted with H) was used for each type of extractive solution. After cooling, the solutions were filtered in a 25 mL volumetric flask and filled to the mark with the same solvent. The extracts were stored at 4 °C until analysis. For each sample, an index corresponding to the solvent with which it was extracted was added, 50% ethanol (*v*/*v*)—indexed by a, 20% ethanol (*v*/*v*)—indexed by b, and dis+9/tilled water—indexed by c. Ethanol was purchased from Sigma-Aldrich (Darmstadt, Germany).

The dosage of flavonoids was measured by a method based on the complexation reaction with AlCl_3_ [41], for phenolic acids based on the property of forming colorimetric oximes [41], and for total polyphenols with the Folin–Ciocâlteu reagent [41,42]. All chemicals for spectrophotometric determinations (aluminum chloride, chlorogenic acid, ethanol, hydrochloric acid, rutin, sodium hydroxide, sodium nitrite, tannic acid, Folin–Ciocâlteu reagent, sodium carbonate) were purchased from Sigma-Aldrich (Darmstadt, Germany).

#### 4.2.1. Quantitative Analysis of Flavonoids (FC)

In a series of 10 mL volumetric flasks, different volumes of the extractive solution were poured: 0.2, 0.4, 0.6, 0.8, and 1 mL; then, 2 mL of sodium acetate 100 g/L and 1 mL of aluminum chloride solution 25 g/L were added and filled to the mark with the same solvent used for extraction. In parallel, the control samples were prepared by measuring 0.2, 0.4, 0.6, 0.8, and 1 mL of the extractive solution and filling up to the mark with the same solvent. After 45 min, the absorbance reading was performed at λ = 427 nm, calibrating each time with the blank sample. The absorbance value must be between 0.1511 and 1.1300 (the range respects the Lambert–Beer law). FC of the extract was expressed in rutin equivalents (mg REs)/g dry herbal product. All spectrophotometric measurements were performed using a JASCO V-530 UV-VIS (Tokyo, Japan) spectrophotometer.

#### 4.2.2. Quantitative Analysis of Phenolic Acids (PAC)

In a series of 10 mL volumetric flasks, different volumes of the extractive solution were poured: 0.2, 0.4, 0.6, 0.8, and 1 mL. Next, 2 mL of 0.5 M hydrochloric acid, 2 mL of Arnow reagent, and 2 mL of 85 g/L sodium hydroxide were added and filled to the mark with distilled water. In parallel, the control samples were prepared under the same conditions but without the Arnow reagent. After 5 min, the absorbance reading was performed at λ = 525 nm, calibrating each time with the blank sample. The absorbance value must be between 0.1923 and 0.9546 (the range respects the Lambert–Beer law). PAC of the extract was expressed in chlorogenic acid equivalents (mg ChAEs)/g dry herbal product.

#### 4.2.3. Quantitative Analysis of Total Polyphenols (TPC)

In a series of 10 mL volumetric flasks, different volumes of the extractive solution (stock solution = 1:10) were poured: 0.4, 0.6, 0.8, and adjusted to 1 mL by adding distilled water. Then, after a few minutes, 1 mL of Folin–Ciocâlteu reagent was added and filled to the mark with 200 g/L sodium carbonate solution. In parallel, the control samples were prepared under the same conditions but without the analyzed solution. After 40 min in the absence of light, the absorbance reading was performed at λ = 763 nm, calibrating each time with the blank sample. The absorbance value must be between 0.2314 and 0.9752 (the range respects the Lambert–Beer law). TPC of the extract was expressed in tannic acid equivalents (mg TAEs/g) dry herbal product.

The following calibration curves were used to determine the phytochemical compounds content: rutin (linearity range: 5.0–35.0 µg/mL, R^2^ = 0.9998, n = 11), chlorogenic acid (linearity range: 11.3–52.7 µg/mL, R^2^ = 0.9998, n = 6), and tannic acid (linearity range: 2.0-12.0 μg/mL, R^2^ = 0.9990, n = 10). The calibration curves are included in the Supplementary Materials Figures S1–S3).

### 4.3. Obtaining and Characterizing the Dry Extract

Hundred g of dried vegetable product (after soaking the vegetable product in approximately 50–100 mL of 50% ethanol (*v*/*v*)) was refluxed with 1.5 L of 50% (*v*/*v*) ethanol for 30 min. A new amount of 50% (*v*/*v*) ethanol 1 L was added and refluxed for another 30 min. Two reflux extractions were performed. After cooling and filtering, the extractive solution obtained was homogenized and subjected to concentration up to 200 mL on a rotary evaporator (Buchi R-215 with vacuum controller V-850, Marshall Scientific, Hampton, VA, USA). The dry extract was obtained by lyophilization (lyophilizer Christ Alpha 1-2 B (Braun Biotech International GmbH, Melsungen, Germany) equipped with vacuum pump RZ 2.5).

#### 4.3.1. Spectrophotometric Determination of Flavonoids, Phenolic Acids, and Total Polyphenols

For spectrophotometric analyses, 0.1 g of dry extract was solubilized in 25 mL of 50% (*v*/*v*) ethanol. The contents of flavonoids, phenolic acids, and total polyphenols were determined using spectrophotometric methods under the same conditions as previously described.

Flavonoids content of the extract was expressed as mg rutin equivalents (mg REs)/g (dw).

Phenolic acid content of the extract was expressed as mg chlorogenic acid equivalents (mg ChAEs)/g (dw).

Total polyphenol content of the extract was expressed as mg tannic acid equivalents (TAEs)/g (dw).

#### 4.3.2. Identification and Quantification of Polyphenol Content by UHPLC-HRMS/MS

Materials used: analytical standards (Sigma-Aldrich, Germany), methanol, ethanol, formic acid (98–100%), and ultrapure water (Merck, Bucharest, Romania). Stock solutions of 1000 mg/L, prepared by dissolving the standard substance in a suitable solvent (acethonitrile, methanol, dimethylformamide), were kept at −20 °C. Individual stock solutions were used to prepare a mixed stock solution of 4 µg/mL. By diluting the volume corresponding to the stock solution mixture in methanol, the desired concentrations were obtained in the range of 0.05–1 μg/mL for plotting the standard curve. The working solutions were stored at −20 °C for a period of one month.

Although the solvent used for the extraction was ethanol, for the analysis of these samples, methanol was used as the solvent; methanol does not significantly influence the results from a qualitative/quantitative point of view.

The parameters specific to the analysis, LC parameters, and MS parameters, are presented in the Supplementary Materials.

For compounds for which analytical standards were not available but were expected to be found in the extracts based on the literature, a list of masses was compiled, and these were identified in total ion current (TIC) by manual search. However, for unknown compounds, the most reasonable molecular formula with a lower mass error was searched in the Chemspider database (www.chemspider.com accessed on 1 October 2023). Considering that flavones, isoflavones, and phenolic acids have many structural similarities, the ion fragments resulting from MS-MS analysis were used to confirm the chemical structure of the identified compounds using the NORMANMassBank spectral databases (https://massbank.eu/MassBank accessed on 1 October 2023), mzCloudeTM, Advanced-Mass (https://www.mzcloud.org/ accessed on 1 October 2023), and PubChem (https://pubchem.ncbi.nlm.nih.gov/ accessed on 5 November 2023). ACDLabs MS Fragmenter 2019.2.1 software (https://www.acdlabs.com/products/adh/ms/index.php accessed on 1 October 2023) was used to generate a fragmentation model of the identified compounds for comparative analysis.

Quantification method validation, quantitative method validation, and optimization of the UHPLC and MS conditions are presented in the Supplementary Materials (Tables S1 and S2).

### 4.4. Evaluation of Antioxidant Activity

The antioxidant activity of the extract was determined on the basis of the scavenging capacity of the free radical ABTS^•+^ (2,2’-azinobis-3-ethylbenzothiazoline-6-sulfonic acid), the DPPH radical (2,2-diphenyl-2-picryl-hydrazyl), and the ferric-reducing capacity (FRAP). The antioxidant capacity was evaluated based on the value of IC_50_ (µg/mL) (the concentration of the extract that inhibits free radical activity (DPPH and ABTS) by 50%) and EC_50_, the concentration at which the absorbance has a value of 0.5 for the ferric reduction capacity. The lower the IC_50_ or EC_50_ value, the more pronounced the antioxidant effect.

#### 4.4.1. Evaluation of the Scavenger Capacity of the DPPH Radical

The method involved the reduction of the 2,2-diphenyl-2-picryl-hydrazyl radical, a stable, violet nitric radical, with the formation of the corresponding yellow hydrazine, whose concentration is proportional to the concentration of the antioxidant. The DPPH method was used according to Ohnishi M et al. with slight modifications [43]. DPPH and ethanol were purchased from Sigma-Aldrich, Germany. For the experimental determinations, 0.1 mM DPPH solution was prepared daily and stored in the dark.

The absorbance value of the DPPH solution ranged between 0.9 and 1 read at λ = 515 nm. A 0.1033 g sample of extract was solubilized in 25 mL ethanol 50% (*v*/*v*) filled to the mark in the volumetric flask. Volumes of 0.1, 0.2, 0.3, 0.4, 0.7, 1, 1.5, 2, 2.5 mL of the previously obtained solution were poured into volumetric flasks of 10 mL and filled to the mark with 50% (*v*/*v*) ethanol. Each 0.5 mL of the solution corresponding to the extracts was treated with 3 mL of 0.1 mM DPPH ethanolic solution, stirred, and stored in the dark for 30 min (respectively, 60 and 90 min). The absorbances were measured at λ = 515 nm, against ethanol, which was used as a blank. In order to control the accuracy, ascorbic acid (Sigma-Aldrich, Germany) was used as a reference for the calibration curve in the range of concentration between 2 and 22 μg/mL (Supplementary Materials, Figure S4).

The formula used to calculate the inhibition (%) of DPPH radical activity is shown in the Supplementary Materials.

To evaluate the scavenger capacity of the DPPH radical, the equations of the calibration lines, inhibition (%) vs. concentration (µg/mL), for the dry extract at 30, 60, and 90 min and the IC_50_ values were determined.

#### 4.4.2. Evaluation of the Scavenger Capacity of the ABTS^•+^ Radical

The ABTS^•+^ radical, in the presence of an antioxidant substrate, loses its blue coloration, and the color changes are accompanied by a decrease in absorbance [44]. For our determination, we chose a reaction time of 6 min, which is frequently used. The antioxidant activity was evaluated according to the method described by Re R. et al. (1999) [45]. ABTS^•+^ ammonium salt and ethanol were purchased from Sigma-Aldrich, Germany, and potassium persulfate was purchased from Merck, Germany. The ABTS^•+^ radical was obtained following the reaction between the ammonium salt of 2,2-azino-bis(3-ethyl-benzothiazoline-6-sulfonic acid) (7.4 mM solution) and potassium persulfate (2.6 mM solution mM) [45], the mixture being stored in the absence of light and in contact for 16 h. A volume of 2 mL of the obtained solution was diluted in a 100 mL volumetric flask with absolute ethanol, so that the absorbance at λ = 734 nm was 0.700 ± 0.02. An amount of 0.1024 g of the extract was solubilized in 25 mL ethanol 50% (*v*/*v*) filled to the mark in the volumetric flask. Volumes of 0.05, 0.2, 0.4, 0.6, 0.8, 1 mL of the previously obtained solution were poured into a 10 mL volumetric flask and filled to the mark with 50% ethanol (*v*/*v*).

For the actual determination, 0.5 mL of the solutions were treated with 3 mL of ABTS^•+^ ethanolic solution, stirred, and stored in the dark for 6 min. The absorbance of the samples was measured at the maximum absorbance of the ABTS^•+^ solution (λ = 734 nm) compared with absolute ethanol. The reduction in the absorbance values represents the inhibition of the ABTS^•+^ solution and is calculated according to the equation presented in the Supplementary Materials.

To evaluate the scavenger capacity of the ABTS^•+^ radical, the equations of the calibration lines, inhibition (%) vs. concentration (µg/mL), for the dry extract at 6 min, with three repetitions, were used to determine the IC_50_ values.

#### 4.4.3. Assessment of Antioxidant Capacity Based on Ferric-Reducing Capacity (FRAP)

The ferric reduction capacity was determined using the modified FRAP [46] method. Potassium ferricyanide (Fe^3+^) is reduced to potassium ferrocyanide (Fe^2+^) under the action of antioxidant compounds and reacts with ferric chloride to form a complex, whose absorbance can be determined at an absorption maximum of 700 nm, with a color shift from orange to blue. A 0.1020 g extract was solubilized in 25 mL ethanol 50% (*v*/*v*) filled to the mark in the volumetric flask. Volumes of 0.1, 0.4, 0.5, 0.7, 1, 1.5, 2, 2.5 mL of the previously obtained solution were poured into 10 mL volumetric flasks and filled to the mark with 50% ethanol (*v*/*v*). From each dilution, 2.5 mL was taken and placed in test tubes, 2.5 mL phosphate buffer pH 6.6 (Sigma-Aldrich, Germany) and 2.5 mL K_3_ (FeCN)_6_ 1% (Sigma-Aldrich, Germany) were added, and the mixture was kept in a water bath at 50 °C for 20 min. Next, 2.5 mL of 10% trichloroacetic acid solution (Sigma-Aldrich, Germany) was added to each sample. Then, 2.5 mL of solution from each test tube was placed in other test tubes, over which 2.5 mL of water and 0.5 mL of FeCl_3_ 0.1% (Sigma-Aldrich, Germany) were added. After 10 min., the absorbance of the samples was measured at λ = 700 nm against a blank obtained from 5 mL of water and 0.5 mL of 0.1% FeCl_3_.

The antioxidant capacity was determined using the EC_50_ value (µg/mL), which represents the concentration of the solutions to be analyzed at which the absorbance has a value of 0.5. The EC_50_ value (µg/mL) was determined from the equation of the regression line, concentration (µg/mL) vs. absorbance, at 30, 60, and 90 min.

### 4.5. Determination of Cytotoxicity

#### 4.5.1. Determination of the In Vivo Cytotoxicity of *Artemia* sp. Larvae (BSLA—Brine Shrimp Lethality Assay)

The BSLA test for the assessment of cytotoxicity in *Artemia salina* larvae is one of the fastest and most effective methods because of the sensitivity of the larvae to a variety of chemical substances [47,48,49]. The test is considered a useful tool for evaluating the preliminary toxicity of plant or animal extracts [50] and is an efficient, cheap, and relatively fast way to detect toxic compounds, requiring only small amounts of sample, e.g., less than 20 mg [51,52].

The evaluation of the cytotoxic activity of the extract was assessed using in vivo assay, the BSLA test; it was quantified by the mortality rate of the *Artemia salina* (L.) larvae. The BSLA test was performed according to the EBPI protocol (Environmental Bio-detection Products) with modifications regarding the control sample and according to the toxicity protocol (Artoxkit M) obtaining larvae by incubating *Artemia salina* eggs (JBL ARTEMIO cysts) in artificial seawater. We would also like to specify that the larvae from this test offered the possibility of studying the organism with high growth with the undifferentiated cell characteristics. Also, in the first hours of life, the tested substance effects were correlated with cytotoxicity, without interferences with digestive enzymes, because the larvae did not require feeding [53].

The conditions of the experiment: the testing was carried out in a static system in microplates, adapted according to the ARTOXKIT protocol, with a volume of 1 mL, at t = 21 °C, pH = 7–7.2, saline water 5‰.

Obtaining *Artemia* larvae: *Artemia salina* larvae were obtained by introducing the cysts into 35‰ saline solution for 24 h under conditions of continuous lighting and aeration. After hatching, larvae in larval stage I (instar I) were separated and placed in experimental vessels, Plexiglas plates with wells (total volume of 1 mL), in saline solutions of 5‰ (Figure S10 in the Supplementary Materials).

The evaluation was carried out with a stereomicroscope to record the effects, and for the analysis of details such as checking the stages or highlighting the development of the larvae, microscopic preparations were made. Organisms exposed to different concentrations of dry extract were evaluated; the movements and passage of larvae in stages II and III were followed, and mortality was recorded after 24 h of exposure, which was the response quantification parameter.

The following concentrations were evaluated: C_1_ = 200 µg/mL; C_2_ = 300 µg/mL; C_3_ = 400 µg/mL; C_4_ = 700 µg/mL; C_5_ = 1001 µg/mL; C_6_ = 1502 µg/mL; C_7_ = 2003 µg/mL; C_8_ = 2503 µg/mL; C_9_ = 2803 µg/mL; C_10_ = 3404 µg/mL; and C_11_ = 4005 µg/mL. For each concentration, 4 repetitions were performed. For control, test enclosures were used without extract, only in saline water or in saline water with 50% ethanol (*v*/*v*) (the solvent used for extraction), corresponding to the ratio used in the test. For statistical interpretation, the following software was used: StatPlus:mac Pro Build 8.0.4.0./Core v.7.8.11, AnalystSoft Inc. (Brandon, FL, USA). Statistical analysis program for macOS. Version v8.

#### 4.5.2. *Daphnia* Species Toxicity Assay

The bioassays were performed according to the described protocol, with some modifications. Briefly, daphnids from the species *Daphnia magna* and *Daphnia pulex* were selected from parthenogenetic cultures prior to the assay. The bioassay was conducted using 12-well tissue culture plates (Greiner Bio-One), with each well housing 10 organisms. The extract was tested at six different concentrations, ranging from 50 to 1000 μg/mL, with each test being duplicated, and an untreated control was used as a negative control. The samples were maintained at constant temperature and humidity (25 °C, 75% RH) in a climatic chamber (MLR-351H; Sanyo). Lethality was observed at the 24 and 48 h marks, and the LC_50_ and 95% confidence interval (CI95%) of the LC_50_ values for each sample were calculated using the least square fit method, facilitated by GraphPad Prism v 5.1 software.

##### *Daphnia magna* Embryo Developmental Assay

The embryo test was performed according to the described protocol in the literature [54] in 12-well tissue culture plates (Greiner Bio-One, Kremsmünster, Austria), with each well containing five embryos. The extract was tested in three concentrations: 50, 500, and 1000 µg/mL. The concentrations were selected following the results obtained in the toxicity assay, and the test was performed against the untreated control. For each sample, two replicates were performed, and the results were compared with an untreated control in the conditions described in the Toxicity Assay section. Every 24 h, the embryos were subjected to microscopic examination (Euromex bScope, Arnhem, The Netherlands) in order to identify the developmental stages and abnormalities.

### 4.6. Prediction of Biological Activity for Selected Phytochemicals

The biological activities of the phytoconstituents that were identified in higher quantities were predicted using three web-servers: PASS (Prediction of Activity Spectra for Substances), SwissTargetPrediction, and SEA (Similarity Ensemble Approach). PASS is a predictive algorithm based on Level 2 Multilevel Neighborhoods of Atom descriptors that uses a Bayesian approach to estimate for input ligands the probability of being active (Pa) or inactive (Pi) on a specific target or the probability of showing a certain therapeutic effect [55]. SwissTargetPrediction is a tool that predicts the probability of interacting with target proteins for small-molecule ligands based on a combination of 2D and 3D similarity measures between query molecules and known active ligands [56]. SEA is an approach that relates proteins based on the chemical similarity of known ligands and calculates Tanimoto similarity scores (Tc) for each query ligand based on 2D topological Daylight fingerprints [57]. SMILES codes of the selected polyphenolic compounds were used as input variables for each approach. The results were manually filtered in order to select key therapeutic targets relevant to neurological disorders.

### 4.7. Molecular Docking Studies

Molecular docking studies were performed to further investigate the potential interactions between the selected polyphenols and previously predicted protein targets. Based on PASS, SwissTargetPrediction and SEA results, we selected CDK5 (cyclin-dependent kinase 5) and GSK-3β (glycogen synthase kinase-3β) for molecular docking experiments. Crystal structures of human CDK5 in complex with activator p25 and a naphthyridine inhibitor (PDB ID: 7VDR, 2.55 Å resolution [58]) and human GSK-3β in complex with a pyrazine inhibitor (PDB ID: 6HK3, 2.35 Å resolution [59]) were retrieved from the RCSB PDB database. Prior to docking, macromolecule structures were prepared using the YASARA structure [60] as follows: water, solvent molecules, and ligands were removed, structural errors were corrected, side-chains were protonated according to the physiological pH (7.4), the hydrogen-bonding network was optimized, and the conformation was optimized by energy minimization with a YASARA2 forcefield.

Ligands were processed by generating 3D coordinates with DataWarrior 5.2.1 [61], followed by energy minimization using the MMFF94s+ forcefield, and protonated according to the physiological pH. Docking simulations were performed using AutoDock Vina v1.1.2 algorithm [62]. The docking search space was set to include the ATP-binding site of both kinases. The docking protocol was validated by docking the co-crystallized inhibitors into the binding site, followed by the determination of RMSD (root mean square deviation) values after superposition of the predicted poses on the experimental coordinates. The docking results were retrieved as predicted binding poses, binding energies (ΔG, kcal/mol), and ligand efficiencies (LE, ΔG/no. of heavy atoms). The predicted interactions between the ligands and target proteins were assessed using UCSF ChimeraX v1.6.1 [63] and BIOVIA Discovery Studio Visualizer (BIOVIA, Discovery Studio Visualizer, Version 17.2.0, Dassault Systèmes, 2016, San Diego, CA, USA).

### 4.8. Statistical Analysis

First, descriptive statistics were performed, and all data are expressed as mean ± standard deviation. Before applying any statistical tests, key conditions were evaluated for each set of experimental data, such as data normality and variance homogeneity. Normality of the data was assessed using the Shapiro–Wilk test. For non-normally distributed data, we transformed them (using base the 10 logarithm) to achieve normality before conducting statistical tests.

To identify significant differences between data groups, a one-way ANOVA was employed with a Tukey post hoc test for multiple comparisons with statistical significance at a 95% confidence level (*p* < 0.05). Principal component analysis (PCA) with Pearson’s correlation coefficient was performed to identify the relationships between phytochemical compounds and antioxidant capacity.

Clustering analysis was conducted to create groups with similar behavior within and differences between groups. Hierarchical cluster analysis (HCA) was used to identify homogeneous compound groups within each extract based on the exact mass, adduct ion (*m*/*z*)/monitored negative ion, and retention time. The “silhouette” method was used to determine the optimal number of groups.

All statistical analyses of the data obtained were performed using GraphPad Software 9, Inc., San Diego, CA, USA, and XLSTAT for Excel 2021 version (Addinsoft, New York, NY, USA).

## 5. Conclusions

In conclusion, according to the performed analysis, it has been shown that *Ajuga chamaepitys* (L.) Schreb., which is widespread in the spontaneous flora of Dobrogea, Romania, can be an important source of polyphenols with potential inhibitory activity on specific targets in neurodegenerative diseases and neuropathic pain. Further studies are warranted to confirm the predicted molecular mechanisms of action and to further investigate the therapeutic potential in animal models of neurological disorders.

## Figures and Tables

**Table 1:** Results of the spectrophotometric determinations for extractive solutions.

Solvent Used for Extraction	Sample	FC Rutin Equivalents (mg REs)/g Dry Herbal Product	PAC Chlorogenic Acid Equivalents (mg ChAEs)/g Dry Herbal Product	TPC Tannic Acid Equivalents (mg TAEs)/g Dry Herbal Product)
50% ethanol (*v*/*v*)—a	aH	16.986 ± 2.846	15.843 ± 4.793	33.180 ± 2.700
20% ethanol (*v*/*v*)—b	bH	12.947 ± 2.343	21.441 ± 5.397	29.470 ± 1.585
H_2_O (*v*/*v*)—c	cH	13.573 ± 3.420	14.580 ± 4.088	26.204 ± 3.184

Notes. Results are presented as the mean ± SD (standard deviation) (*n* = 3). Legend: H (aerial parts samples), FC = flavonoids content; PAC = phenolic acids content; TPC = total phenolic content. For each sample, corresponding index to the solvent extracted was added, 50% ethanol (*v*/*v*)—a, 20% ethanol (*v*/*v*)—b and distilled water—c.

**Table 2:** Phytochemical compounds identified in ACHE by UHPLC-HRMS/MS.

ACHE—19 Identified Compounds				
Identified Compound	Chemical Formula	Exact Mass	Adduct Ion (*m*/*z*) (Monitored Negative Ion)	Retention Times (Rt-min)
**Flavonoids (Flavan-3-Ols, Flavones, Flavonols, Flavanones, Heterosides)**				
apigenin-7-O-glucosylglucoside	C_27_H_30_O_15_	594.15847	593.15121	17.9
kaempferol-3-O-rutinoside	C_27_H_30_O_15_	594.15847	593.15122	17.9
apigetrin (apigenin-7-glucoside)	C_21_H_20_O_10_	432.10564	431.09839	20.19
vitexin (apigenin-8-C-glucoside)/isovitexin	C_21_H_20_O_10_	432.10564	431.09839	20.19/21.37
kaempferol (or luteolin)-O-glucoside/isomers	C_21_H_20_O_11_	448.10056	447.09331	20.31
cynaroside (luteolin-7-O-glucoside)	C_21_H_20_O_11_	448.10056	447.0932842	20.31
apigenin-7-O-glucuronide	C_21_H_18_O_11_	446.08491	445.0776342	21.23
naringenin	C_15_H_12_O_5_	272.06847	271.06122	22.71
kaempferol	C_15_H_10_O_6_	286.04774	285.04049	23.2
luteolin	C_15_H_10_O_6_	286.04774	285.0404862	23.87
apigenin	C_15_H_10_O_5_	270.05282	269.04502	24.11
hispidulin	C_16_H_12_O_6_	300.06339	299.0561362	24.28
chrysin	C_15_H_10_O_4_	254.05791	253.05066	25.72
2’,6-dihydroxyflavone	C_15_H_10_O_4_	254.05791	253.05066	25.72
**Isoflavones**				
genistin	C_21_H_20_O_10_	432.10565	431.09837	19.64
pratensein	C_16_H_12_O_6_	300.06339	299.05614	24.28
biochanin A	C_16_H_12_O_5_	284.06847	283.06122	26.21
**Phenolic acids**				
chlorogenic acid	C_16_H_18_O_9_	354.09508	353.08783	10.64/13.86
caffeic acid	C_9_H_8_O_4_	180.04226	179.03501	14.48

**Table 3:** Polyphenolic compounds quantified in plant extract by UHPLC-HRMS/MS.

Compound	µg/g Dry Extract
caffeic acid	3253.8
p-coumaric acid	198.2
syringic acid	84.2
genistin	730.2
ferulic acid	254.3
apigenin	325.7
rutin	110.6
ellagic acid	18.2
pinocembrin	32.7
galangin	283.3
chrysin	90.22
kaemferol	3041.5
naringenin	395.0

**Table 4:** Concentration values at which lethal effects were recorded (at the 95% confidence level).

Lethal Effects	µg/mL
LC_10_	557.85
LC_16_	1006.78
LC_50_	2600.25
Standard error	105.65
Lower 95%	2326.97
Upper 95%	2873.53
LD_84_	4193.72
LD_90_	4642.65
LC_100_	4990.46
**Pearson Chi-Square**	
Chi-square	0.45
Degrees of Freedom	9
*p*-value	1

**Table 5:** Predicted activity on CDK5 and GSK-3β using three different algorithms.

	PASS (Pa)		SwissTargetPrediction (P)		SEA (max Tc)	
Compound	CDK5	GSK-3β	CDK5	GSK-3β	CDK5	GSK-3β
apigenin	0.0780	-	1.0000	1.0000	0.6154	1.0000
caffeic acid	-	-	-	0.0000	-	-
ferulic acid	-	-	-	-	-	-
galangin	-	0.0980	0.1497	0.1661	0.4565	-
genistein	-	-	0.1006	0.1006	-	-
kaempferol	-	0.0690	0.5179	0.6580	-	-
naringenin	-	-	0.1006	0.1006	-	-
p-coumaric acid	0.0690	-	-	0.0000	-	-

Pa, P—probability of being active, max Tc—maximum Tanimoto coefficient.

**Table 6:** Predicted binding energies and ligand efficiencies following docking in the ATP-binding site of CDK5 and GSK-3β.

	CDK5		GSK-3β	
Compound	ΔG (kcal/mol)	LE	ΔG (kcal/mol)	LE
apigenin	−9.315	0.4657	−8.551	0.4275
caffeic acid	−7.663	0.5895	−6.789	0.5222
ferulic acid	−7.261	0.5186	−6.729	0.4806
galangin	−9.039	0.4519	−8.553	0.4277
genistein	−8.909	0.4455	−8.259	0.4129
kaempferol	−8.548	0.4070	−8.337	0.3970
naringenin	−9.232	0.4616	−8.537	0.4268
p-coumaric acid	−7.592	0.6327	−6.625	0.5521

## Data Availability

Data are contained within the article and Supplementary Materials.

## Supplementary Materials

The following supporting information can be downloaded at: https://www.mdpi.com/article/10.3390/plants13091192/s1, Figure S1. Standard curve of rutin. Figure S2. Standard curve of chlorogenic acid. Figure S3. Standard curve of tannic acid. Figure S4. Standard calibration curve for ascorbic acid as a reference in 50% ethanol. Figure S5. Equations of the calibration lines, inhibition (%) vs. concentration (µg/mL) for ACHE in 50% ethanol using the DPPH method (30 min—(a); 60 min—(b); 90 min—(c)). Figure S6. Equations of the calibration lines, inhibition (%) vs. concentration (µg/mL) for ACHE in 50% ethanol using ABTS^•+^ method (6 min (I)—(a); 6 min (II)—(b); 6 min (III)—(c)). Figure S7. Equations of the calibration lines, concentration (mg/mL) (µg/mL) vs. absorbance for ACHE in 50% ethanol using the FRAP method (30 min—(a); 60 min—(b); 90 min—(c)). Figure S8. Principal component analysis (PCA) of phenolic content (TPC, TFC, and FC) and antioxidant determination assays (DPPH, ABTS^•+^, and FRAP). Figure S9. Probit values correlated with the tested concentrations. Figure S10. The test system in microplates with wells (1mL test volume). Figure S11. Regression calculated for the evaluated effects (mortality). Table S1. Validation parameters of the LC-HRMS analytical method. Table S2. Monitored compounds by full-scan HRMS analysis and MS-MS analysis based on analytical standards. Table S3. Correlation between phenolic contents (TPC, FC, and PAC) and antioxidant activities (DPPH, FRAP, and ABTS^•+^) as Pearson’s correlation coefficients (r). References [64,65,66] are cited in the Supplementary Materials.
