# Supplementary material for: Phytochemical, In Vitro, In Vivo, and In Silico Research on the Extract of Ajuga chamaepitys (L.) Schreb
Source: Plants (Basel). 2024 Apr 25;13(9):1192. doi: 10.3390/plants13091192 (PMC11085171; doi:10.3390/plants13091192)
Supplement: Supplementary file 1 [file plants-13-01192-s001.zip › plants-2950763-supplementary.pdf]

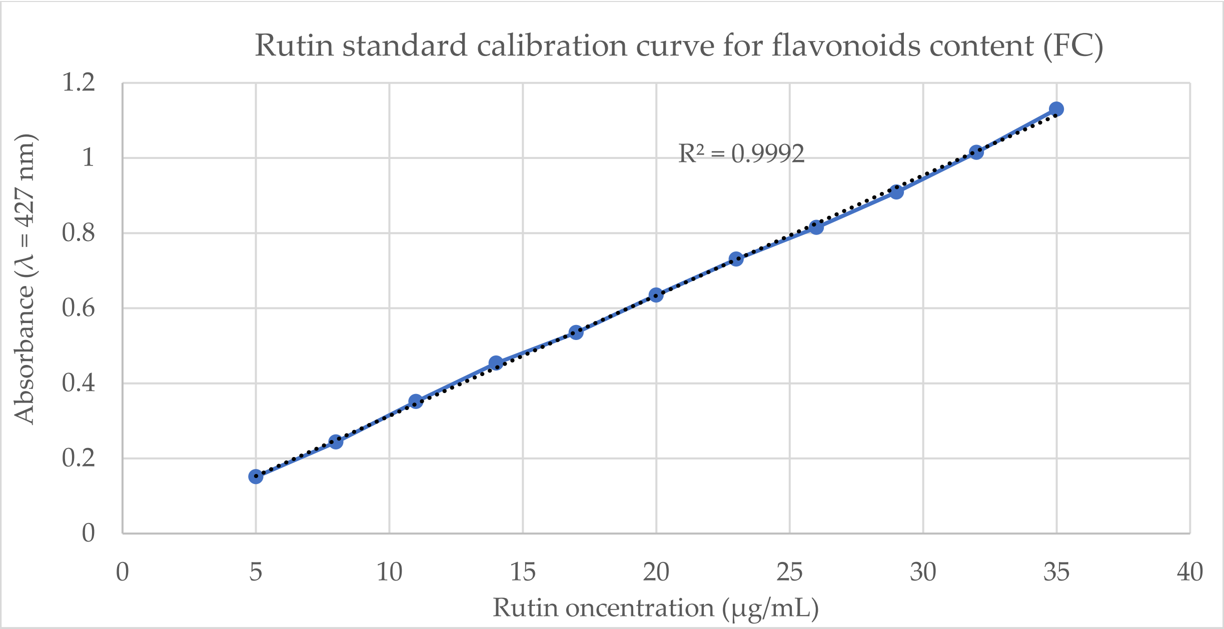

Figure S1. Standard curve of rutin.

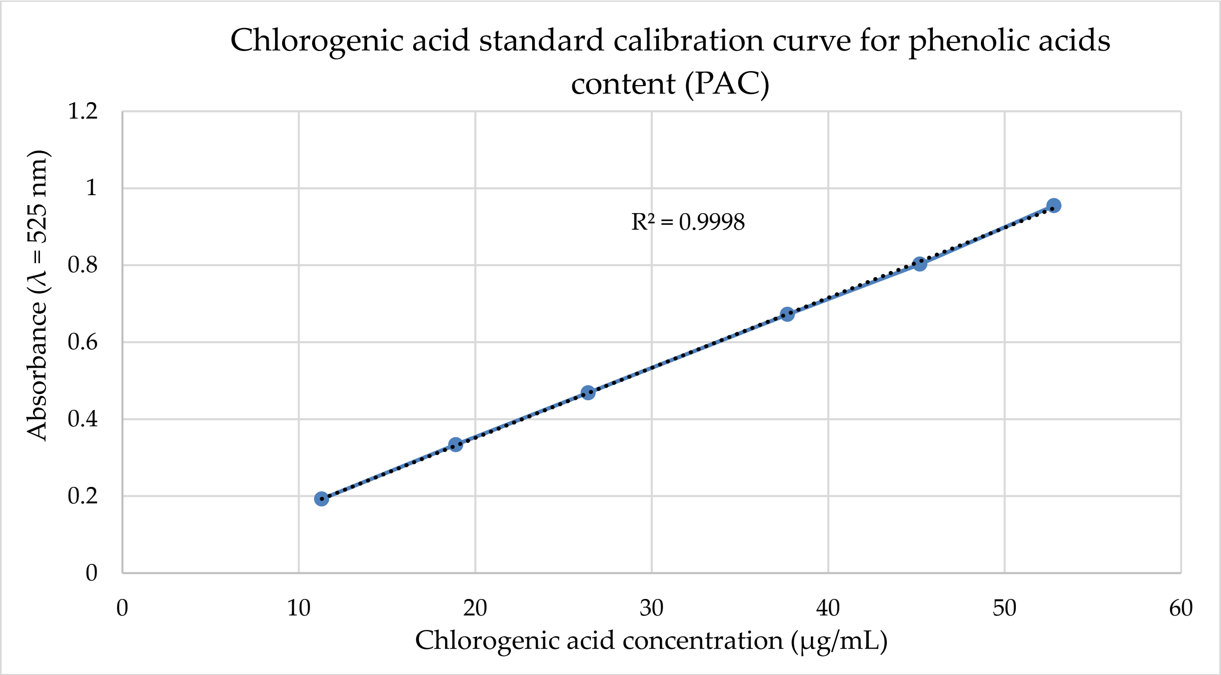

Figure S2. Standard curve of chlorogenic acid.

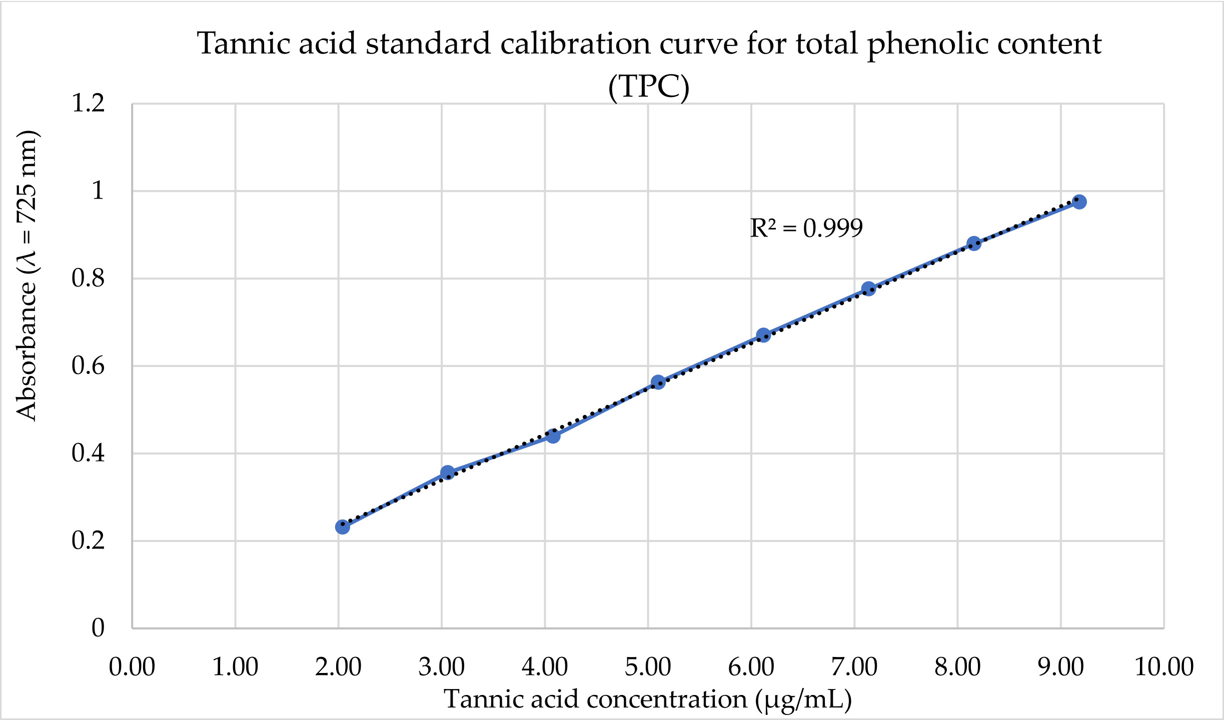

Figure S3. Standard curve of tannic acid.

Optimization of UHPLC and MS conditions

LC parameters. A UHPLC system consisting of a Thermo Scientific Dionex Ultimate 3000 Series RS pump, a Thermo Scientific Dionex Ultimate 3000 Series TCC-3000RS column thermostated compartment, and a Thermo Fisher Scientific Ultimate 3000 Series WPS-3000RS autosampler, controlled by Chromeleon 7.2 Software (Thermo Fisher Scientific, Waltham, MA and Dionex Softron GmbH Part of Thermo Fisher Scientific, Germany). Chromatographic column used: Acquity U-HPLC HSS T3 (100x2.1mm, 1.8pm) (Waters, Etten-Leur, The Netherlands). A flow rate of 0.4 mL min-1 was established for the separation of selected compounds from the UHPLC system. Mobile phase: eluent A, water containing 100 μL formic acid/L water for pH correction to 3.3; eluent B, methanol. The gradient was made as follows: min 0-1 100% A; min 1-10 linear increase at 30% B; min 10–26, linear increase to 100% B and hold 4 min; min 30-32.5 linear decrease to 0% B; hold 1.5 min. The column temperature was set at 45°C, the injection volume at 10 μL and a HESI ion source (Electrospray) was used for negative ionization. HESI parameters were: gas flow - 40 units; auxiliary gas flow - 10 units; capillary temperature: 255°C; auxiliary gas heating temperature: 400°C; spraying voltage -2800 V; objective S: RF level, 50.

MS parameters. Detection of compounds was performed using a Q-Exactive mass spectrometer (Thermo Fisher Scientific). Data acquisition was performed in the vDIA mode, which includes full-scan MS analysis and simultaneous MS/MS analysis, both in negative ionization mode. Full-scan MS analysis was performed at a resolving power of 70 000 FWHM at m/z 200. For the compounds of interest, the scan range chosen was m/z 130 – 1000. The automatic gain control (AGC) was set at 3e6, and the injection time was set to 200 ms. vDIA MS/MS analysis was performed by successive MS2 scan events as follows: precursor ion ranges m/z 95–205, 195–305, 295–405, 395–505, and 500–1005 were consecutively selected, fragmented in the HCD cell, and the resulting ions measured in five separate Orbitrap scans with a resolving power of 35,000 (m/z 200). The automatic gain control (AGC) was set to 1e6, and the injection time was set to 100 ms. Fragmentation was performed using the collision energy in steps between 20% and 60% normalized

collision energy (NCE). Data were analyzed using Quan/Qual Browser Xcalibur 2.3 (Thermo Fisher). The mass tolerance window was set to 5 ppm for the two analysis modes. For comparative purposes, an MS/MS (targeted MS-MS) analysis was performed separately using a mass inclusion list and expected retention times for target analytes for which analytical reference standards were available, with a time window of 30 seconds.

The mobile phase and flow rate were investigated and adjusted for optimal analyte separation. According to previous studies, methanol was chosen as the organic component of the mobile phase because the addition of an organic acid prevents the adaptation of the phenolic hydroxyl fragment to the C18 chromatographic column (La Barbera *et al.*, 2017).

Polyphenolic compounds generally show better mass spectrometric responses to negative ionization than to positive ionization (La Barbera *et al.*, 2017, Ciucure and Geană, 2019). Therefore, 0.05% and 0.1% formic acid aqueous solutions were tested as mobile phase A at a flow rate of 0.3 mL/min. Based on these results, 0.05% formic acid was preferred as mobile phase A. The elution gradient was progressively slowed to increase the number of chromatographic peaks. The ESI ionization parameters were optimized to achieve high sensitivity for most compounds.

Operating in vDIA mode, the instrument alternates between MS and MS2 scans, and sufficient data/points per chromatographic peak are required to ensure a large number of detected peaks.

Validation of the polyphenol quantification method using the UHPLC– HRMS/MS technique

The analytical performance of target HRMS analysis of polyphenolic compounds in samples was expressed in terms of linearity, correlation coefficients (R2), limit of detection (LOD), and limit of quantification (LOQ). Calibration was performed in the concentration range of 0.025 – 2.5 μg/mL for each of the phenolic compounds by serial dilution with a mixture of water : methanol (90 : 10) from the 1 mg/L stock standard mixture. The limit of detection (LOD) and limit of quantification (LOQ) for each target compound were determined to be 3.3-fold and 10-fold, respectively, based on the standard deviation of the y-intercept divided by the slope of the calibration curve.

Method precision was assessed by repeatability and reproducibility analysis and expressed as relative standard deviations (RSD). The acceptance criterion was an RSD value below 5.0 %. Repeatability was assessed by analyzing six samples from the above, and reproducibility was examined for three consecutive days with six samples per day. The results are expressed as μg/g of lyophilized plant extract.

The R2 correlation coefficient for all standards was greater than 0.99, showing good linearity, and the standard deviation (RSD) values of the target compounds were less than 5%, demonstrating good precision and accuracy. The limit of detection (LOD) and limit of quantification (LOQ) values were maintained in the range of 0.10 – 10.50 μg/g DW (dry weight) and 0.30 – 35.10 μg/g DW (dry weight), respectively.

Table S1. Validation parameters of the LC-HRMS analytical method.

**Compounds**	**Calibration equations**	**R^2**	**LOD μg/g DW**	**LOQ μg/g DW**	**Precision (RSD, %)**		**Repeatability (RSD, %)**
					**intra-day**	**inter-day**	
catechin	y=824313.8x+203017.7	0.991	0.40	1.30	3.15	2.85	3.21
epicatechin	y=230287.5x-168641.6	0.992	0.85	2.83	1.41	2.62	2.16
caffeic acid	y=781534.3x+393060	0.990	1.84	6.13	2.36	4.10	3.82
myricetin	y=32881x-738887	0.994	1.21	4.03	0.89	1.36	2.07
p-coumaric acid	y=82377489.4x+486112	0.995	0.62	2.06	1.23	3.04	2.95

syringic acid	y=72565.8x-82305	0.993	0.35	1.16	2.81	4.01	3.87
genistin	y=18732x-110805	0.998	1.65	5.50	1.65	1.85	2.04
chlorogenic acid	y=174329.1x+19222.5	0.998	0.71	2.30	3.45	3.89	4.01
ferulic acid	y=121050x-2321515	0.999	0.15	0.50	1.25	1.87	2.56
hyperoside	y=117573.7x+1512847.8	0.998	0.47	1.56	3.21	4.32	3.58
isorhamnetin	y=3022x+156072	0.999	1.02	3.40	2.47	2.67	2.12
rutin	y=102426.9x+77215.4	0.991	0.65	2.16	2.43	3.16	3.04
gallic acid	y=449675.5x-332050.7	0.994	0.31	1.03	1.26	2.08	2.74
ellagic acid	y=10520x-1437986	0.990	1.03	3.04	1.11	1.15	1.27
formononetin	y=2628478x+16100393	0.999	0.42	1.40	1.48	1.58	2.23
ononin	y=211703.5x+1291087	0.992	10.50	35.10	3.04	4.11	3.98
pinocembrin	y=1301012x-4130476	0.999	0.10	0.30	1.95	2.28	2.65
apigenin	y=897709x-73484551	0.998	0.22	0.73	1.65	2.90	3.07
galangin	y=866716.1x+21534478	0.998	0.15	0.47	1.04	1.87	1.95
pinostrobin	y=695357x-22084171	0.999	0.15	0.50	2.76	3.16	3.82
kaempferol	y=324023.5x+35109689	0.991	0.30	1.00	3.07	3.65	3.18
hesperetin	y=1635.3x-120620	0.990	0.95	3.16	2.89	3.07	3.14
genistein	y=1598102x+5526494	0.999	0.30	1.00	1.58	2.49	2.61
naringenin	y=818977.8x+1767512	0.994	0.14	0.46	2.80	3.74	3.06
naringin	y=73403.1x+53366.8	0.998	6.90	23.00	1.08	2.16	3.85
quercetin	y=125092.1x+747968	0.999	1.60	5.33	1.11	2.65	1.85
glycitein	y=259340.7x+6212357	0.999	0.55	1.83	2.08	3.18	2.74
daidzin	y=1017.2x-74781	0.997	1.08	3.60	2.65	3.74	4.08
daidzein	y=2071x+3009.8	0.999	1.15	3.83	1.43	2.65	2.14
chrysin	y= 430529x-185238	0.995	0.21	0.70	1.08	2.14	2.68
abscisic acid	y=222271x-396923	0.996	0.15	0.37	1.66	1.89	1.54

Table S2. Monitored compounds by full scan - HRMS analysis and MS-MS analysis based on analytical standards.

Compound	Rt (min)	Chemical formula	Exact mass	Error (ppm)	$[M-H]^-$ Ion (m/z)	MS2 Fragment ions (m/z)
			**Flavonoids**			
catechin	7.80	$C_{15}H_{14}O_6$	290.07904	1.47	289.07176	245.08192; 203.07088; 151.03908; 125.02320; 109.02821
epicatechin	10.19	$C_{15}H_{14}O_6$	290.07904	1.25	289.07176	245.08192; 203.07088; 151.03908; 109.02821
quercetin	16.59	$C_{15}H_{10}O_7$	302.04265	0.86	301.03540	245.04601; 178.99809;

						273.04059; 121.02814
rutin (quercetin-3-O-rutinoside)	14.20	$C_{27}H_{30}O_{16}$	610.15338	0.50	609.14613	300.02777; 271.02505; 255.02995; 243.02980; 165.01841; 151.00258
apigenin	17.54	$C_{15}H_{10}O_5$	270.05282	1.18	269.04502	227.03389; 181.06430; 151.00194; 149.00226; 117.03271
kaempferol	17.06	$C_{15}H_{10}O_6$	286.04774	0.57	285.04049	255.02977; 201.01866; 151.00262; 107.01250; 92.92660
isorhamnetin	13.20	$C_{16}H_{12}O_7$	316.05830	1.35	315.05105	300.02710; 227.03508; 163.00369; 151.00264; 107.01190
naringenin	19.69	$C_{15}H_{12}O_5$	272.06847	0.50	271.06122	253.05055; 151.00269; 119.04903; 107.01258
naringin	14.11	$C_{27}H_{32}O_{14}$	580.17920	2.02	579.17185	356.99371; 255.02995; 119.04884
hesperetin	16.83	$C_{16}H_{14}O_6$	302.07904	1.34	301.07179	283.06204; 267.06650; 252.04286; 151.00266; 125.02319
pinostrobin	17.40	$C_{16}H_{14}O_4$	270.08921	1.89	269.08196	254.05864; 210.06839; 177.05495; 148.01559
chrysin	17.63	$C_{15}H_{10}O_4$	254.05791	1.65	253.05066	208.96011; 151.03899; 107.04897; 89.04897; 65.03819
myricetin	10.42	$C_{15}H_{10}O_8$	318.03757	1.37	317.03032	178.99860; 164.92636; 151.00368;

						137.02442; 107.01258
galangin	19.98	$C_{15}H_{10}O_5$	270.05282	1.48	269.04557	239.03345; 227.03389; 225.05580; 211.03877; 169.06425
hyperoside (quercetin-3-galactoside)	13.98	$C_{21}H_{20}O_{12}$	464.09548	1.03	463.08768	300.02771; 355.02985; 271.02491; 243.02969; 178.99773; 151.00262
genistin	12.56	$C_{21}H_{20}O_{10}$	432.10565	1.45	431.09837	311.05637; 269.04590; 271.05133; 181.06580
genistein	18.07	$C_{15}H_{10}O_5$	270.05282	1.24	269.04502	159.04420; 133.02835; 201.05527; 181.06546; 107.01257
daidzin	11.42	$C_{21}H_{20}O_9$	416.11073	1.49	415.10348	252.04510; 251.03490; 224.04870; 223.03980
daidzein	16.50	$C_{15}H_{10}O_4$	254.05791	0.87	253.05066	226.05887; 224.04649; 209.06091; 197.06055; 135.00686; 117.03333
ononin	26.14	$C_{22}H_{22}O_9$	430.12638	2.06	429.11913	355.09690; 341.11090; 267.10280; 252.00778
formononetin	18.74	$C_{16}H_{12}O_4$	268.07356	1.19	267.06631	252.04298; 223.03986; 195.04466; 132.02049
glycitein	16.33	$C_{16}H_{12}O_5$	284.06847	1.18	283.06122	268.03750; 240.04830; 211.03979; 196.05252; 167.02063
			**Phenolic acids**			

gallic acid	1.73	$C_7H_6O_5$	170.02152	0.16	169.01427	125.02318; 141.01823
chlorogenic acid	8.20	$C_{16}H_{18}O_9$	354.09508	0.24	353.08783	192.05876; 191.05544; 173.04474; 127.03876; 85.02806
caffeic acid	8.71	$C_9H_8O_4$	180.04226	0.37	179.03501	135.04390; 107.04881
ferulic acid	14.98	$C_{10}H_{10}O_4$	194.05791	0.62	193.05066	178.02635; 149.05974; 134.03615; 106.04240
ellagic acid	14.44	$C_{14}H_6O_8$	302.00627	1.62	300.99899	185.02349; 283.98961; 229.01391; 157.01006
abscisic acid	15.73	$C_{15}H_{20}O_4$	264.13616	1.42	263.12891	263.12854; 219.13864; 204.11502; 153.09126; 136.05162
p-coumaric acid	10.77	$C_9H_8O_3$	164.04734	0.18	163.03954	211.07640; 135.00754; 119.05020; 17.03320; 116.02670
syringic acid	15.38	$C_9H_{10}O_5$	198.05282	0.41	197.04555	182.02049; 123.00697; 166.99693

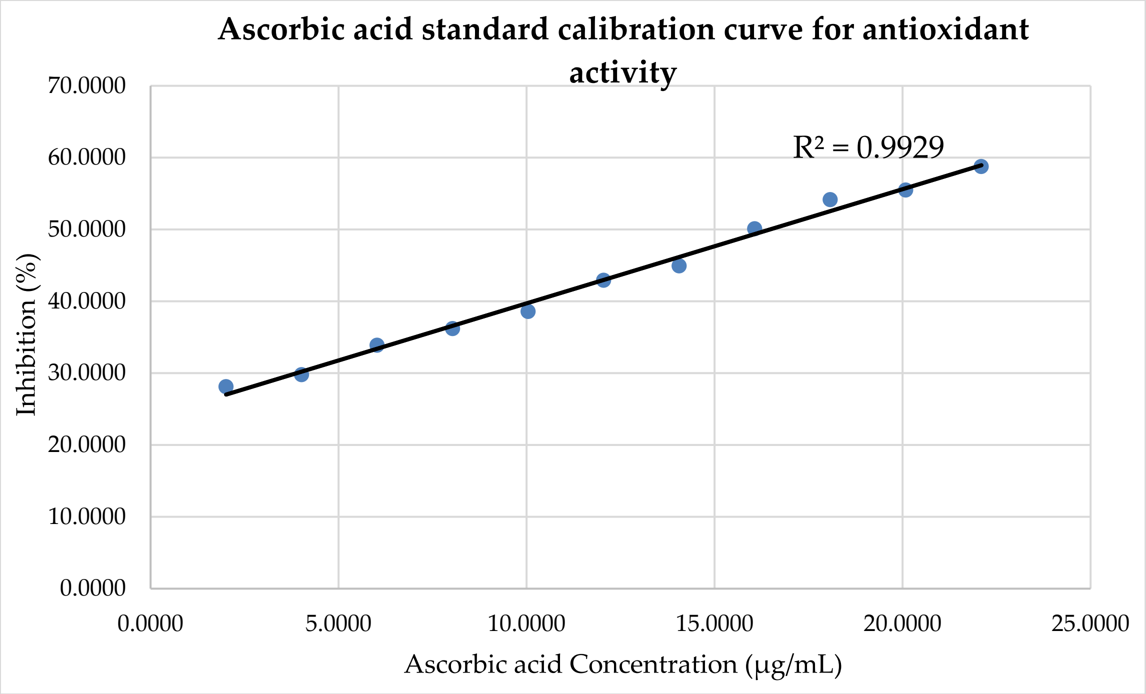

Figure S4. **Standard calibration curve for ascorbic acid as a reference in 50% ethanol**

Evaluation of the scavenger capacity of the DPPH radical.

The inhibition (%) of DPPH radical activity was calculated using the following formula:

$$\%Inhibition_{\text{DPPH}} = \frac{A\,(blank) - A\,(sample)}{A\,(blank)}\ x\ 100$$

where:

$A_{\text{(blank)}}$ = blank absorbance of 0.1 mM DPPH solution in the absence of extract.

$A_{\text{(sample)}}$ = sample absorbance of the DPPH solution in the presence of extract after 30 min, 60 min, and 90 min.

Evaluation of the scavenger capacity of the ABTS•+ radical

$$\%Inhibition_{\text{ABTS}^{\bullet+}} = \frac{A\,(t{=}0min) - A\,(t{=}6min)}{A\,(t\,{=}0min)}\ x\ 100$$

where:

$A_{\text{(t = 0 min)}}$ = absorbance of the blank sample (ABTS•+ sol in the absence of tested compounds: 0.70 ± 0.02);

$A_{\text{(t = 6 min)}}$ = absorbance of the vegetal extract (ABTS•+ sol in the presence of tested compounds).

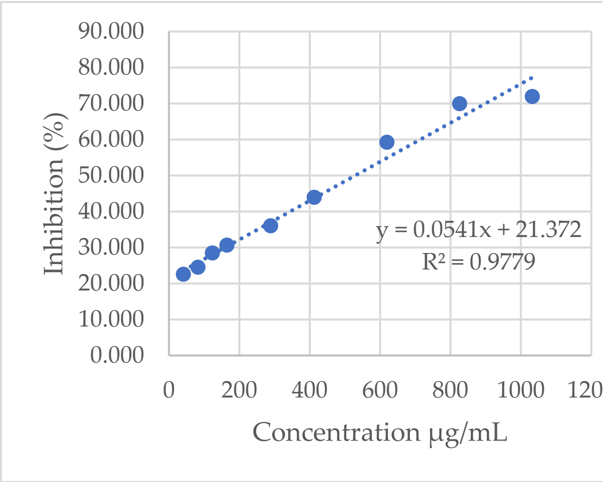

(**a**)

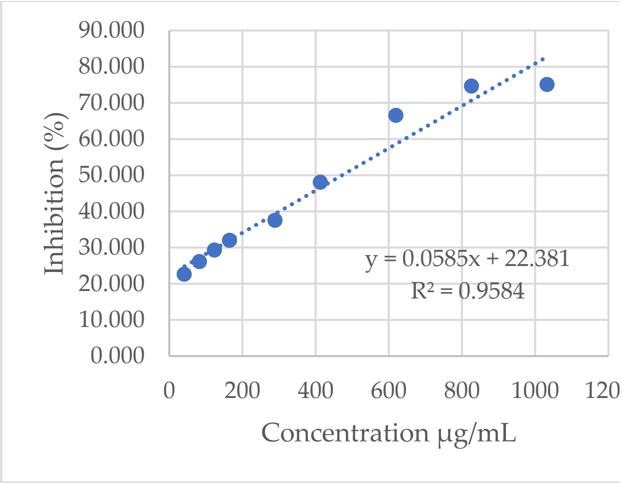

(**b**)

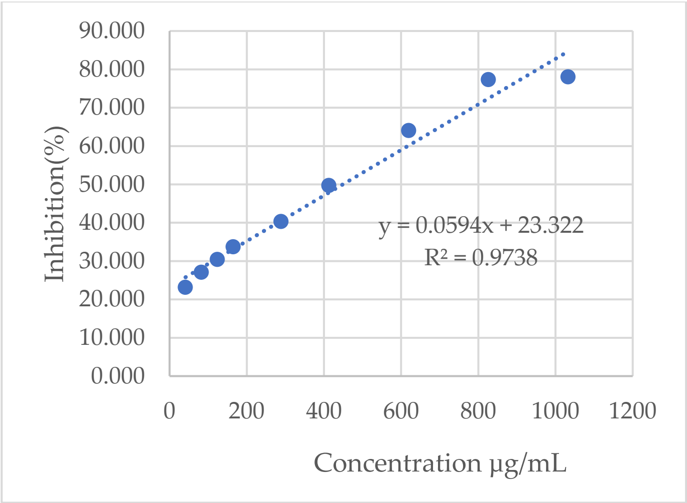

(**c**)

Figure S5. Equations of the calibration curves ~~lines~~, inhibition (%) vs. concentration (~~mg/mL~~) µg/mL for ACHE in 50% ethanol using the DPPH method (30 min – (a); 60 min – (b); 90 min – (c)).

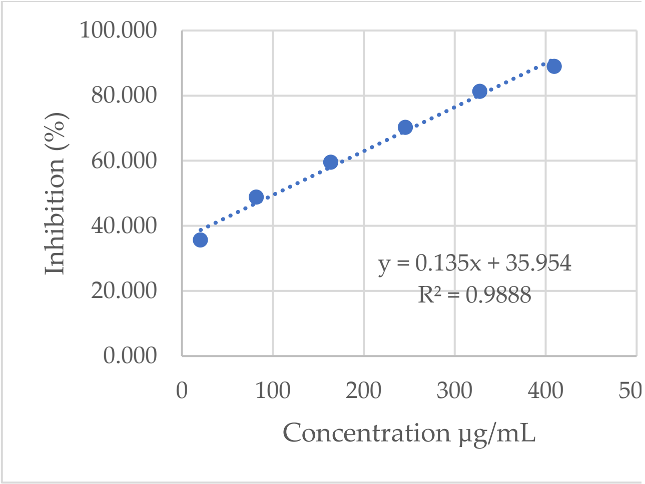

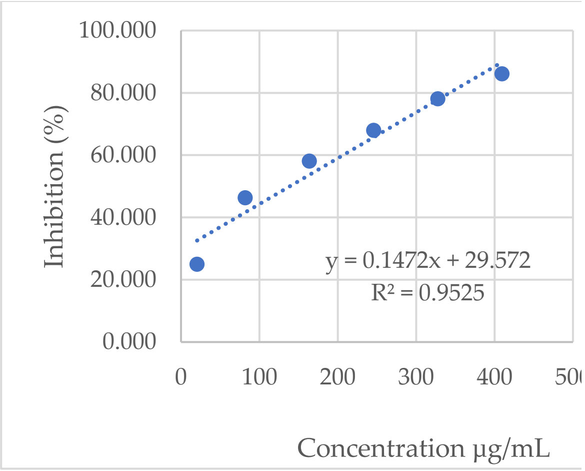

(**a**) (**b**)

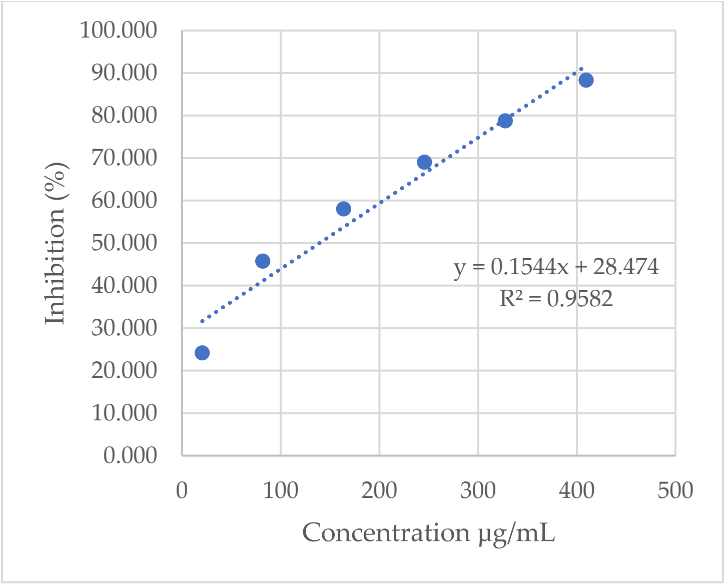

(c)

Figure S6. Equations of the calibration curves ~~lines~~, inhibition (%) vs. concentration ~~(mg/mL)~~ (µg/mL) for ACHE in 50% ethanol using $ABTS^{\bullet +}$ method (6 min (I) – (a); 6 min (II) – (b); 6 min (III) – (c)).

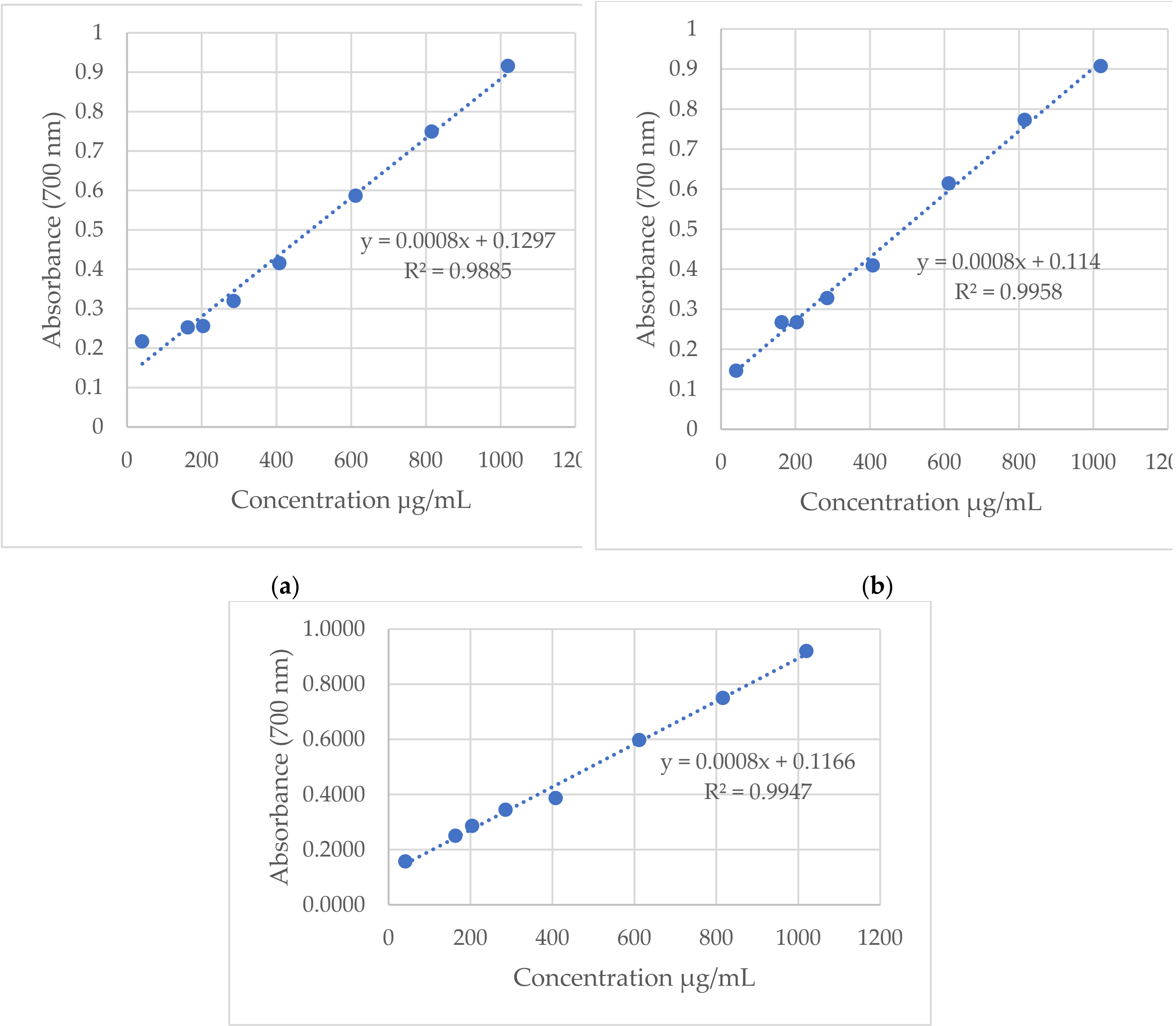

(**a**) (**b**)

(c)

Figure S7. Equations of the calibration curves ~~lines~~, concentration (~~mg/mL~~) (μg/mL) vs. absorbance for ACHE in 50% ethanol using the FRAP method (30 min – (a); 60 min – (b); 90 min – (c)).

The correlation of the phenolic contents (TPC, FC, and PAC) and antioxidant activities (DPPH, FRAP, ABTS$^{•+}$) was evaluated using Pearson's correlation test (Table S3). A positive, strong correlation was observed between DPPH and FC ($r = 0.9959$, $p = 0.009$), PAC ($r = 0.9991$, $p < 0.001$), and TPC ($r = 0.9704$, $p = 0.0367$). A significant negative correlation was found between ABTS$^{•+}$ and FC ($r = -0.9639$, $p < 0.0386$) and PAC ($r = -0.9719$, $p = 0.0249$). No significant correlation was found between FRAP and phenolic contents.

Table S3. Correlation between phenolic contents (TPC, FC, and PAC) and antioxidant activities (DPPH, FRAP, and ABTS$^{•+}$) as Pearson's correlation coefficients (r)

Variables	FC	PAC	TPC	DPPH	FRAP	ABTS
Flavones	**1**					
Phenolic acids	**0.9960**	**1**				
Total polyphenols	**0.9606**	**0.9596**	**1**			
DPPH	**0.9959**	**0.9991**	**0.9704**	**1**		

FRAP	-0.4796	-0.4661	-0.6963	-0.5024	**1**	
ABTS	**-0.9639**	**-0.9719**	-0.8663	**-0.9613**	0.2447	**1**

Values in bold are different from 0 with a significance level alpha=0.05

Additionally, principal component analysis (PCA) was used to study the similarities and differences between methods applied to estimate antioxidant activity and measure the phenolic content (Figure S8). Figure S8 indicates that 99.90% of the total variability present in the original data was captured by the first two factors, F1 (85.81%) and F2 (14.29). DPPH and ABTS$^{•+}$ strongly correlate with PC1 in the positive and negative directions, respectively, while the values of FRAP correlate with PC2 in the positive direction.

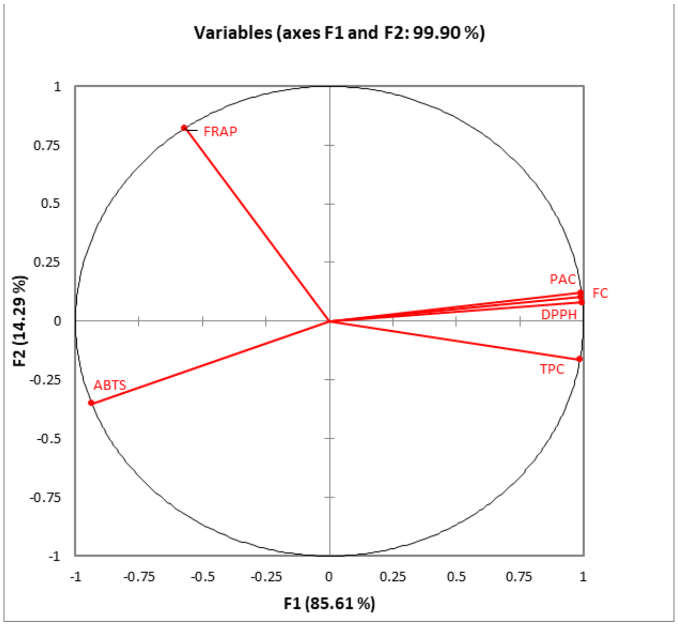

Figure S8. Principal component analysis (PCA) of phenolic content (TPC, TFC, and FC) and antioxidant determination assays (DPPH, ABTS$^{•+}$ and FRAP).

The results obtained were conclusive, the content ~~amount~~ of phytochemical compounds playing a main role in improving the oxidation process by capturing free radicals, chelating metal ions, and eliminating oxygen from biological systems [Lü et al. 2010].

Determination of the *in vivo* cytotoxicity of *Artemia* sp . larvae (BSLA - Brine Shrimp Lethality Assay)

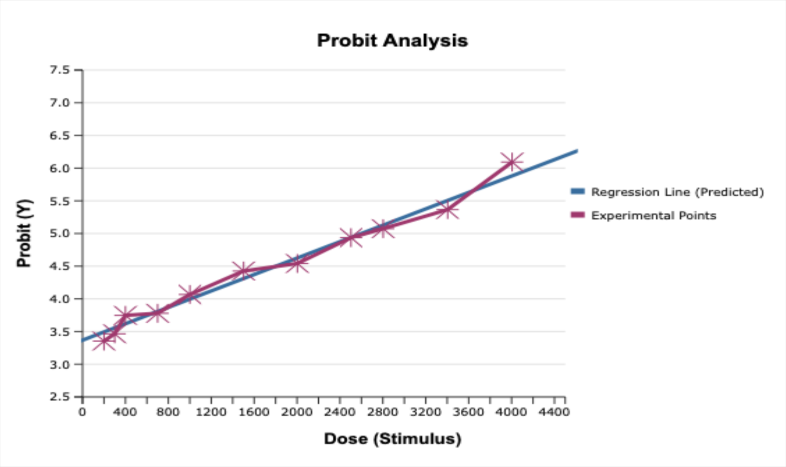

Figure S9. Probit values correlated with the tested concentrations

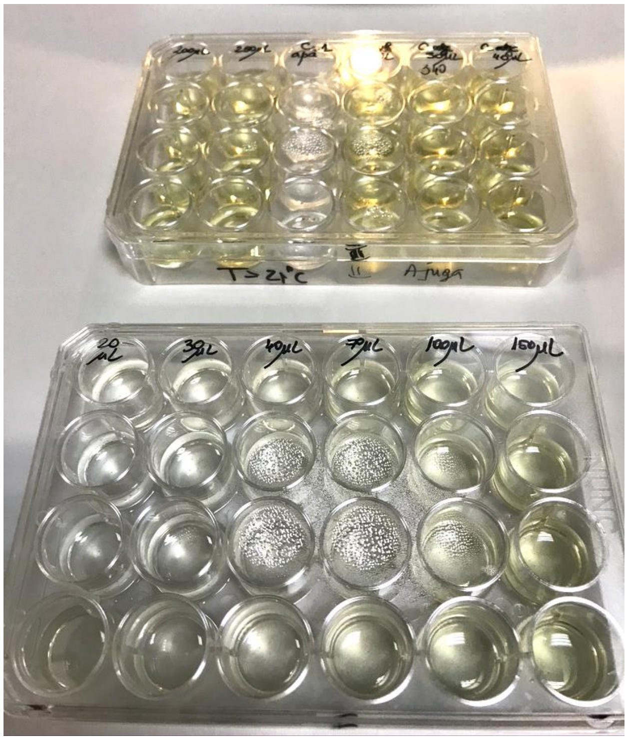

Figure S10. The test system in microplates with wells (1mL test volume)

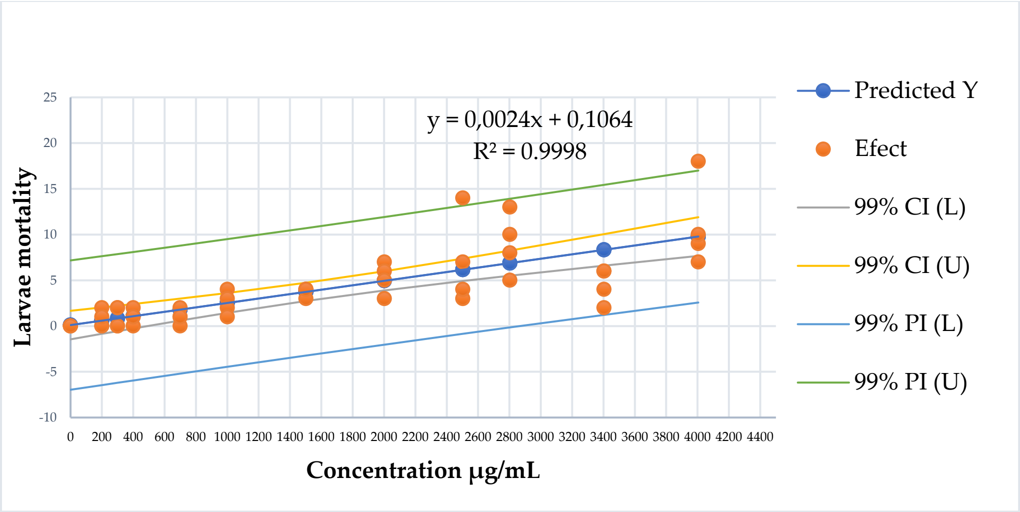

Figure S11. Regression calculated for the evaluated effects (mortality).
